# Hypoxic cancer stem cell–immune niches in the tumor microenvironment: a Systematic Review of mechanisms and therapeutic implications

**DOI:** 10.3389/fimmu.2026.1942424

**Published:** 2026-09-22

**Authors:** Prasanna Appiya Premvignesh, Abubakker Siddiq Mohamed Hameed, Ibrahim Mustufa Topiwala, Mohammed Nayeem Shaji, Latifa Alsaad, Safaa Mahmoud Mohamed Abdelkhalek, Nazeerullah Rahamathullah, Salem Chouaib

**Affiliations:** 1 Department of Biomedical Sciences, College of Medicine, Gulf Medical University, Ajman, United Arab Emirates; 2 Faculty of Medicine, Medical University of Lublin, Lublin, Poland; 3 Research and Graduate Studies, Mohammed Bin Rashid University of Medicine and Health Sciences, Dubai, United Arab Emirates; 4 Department of Pathology, Faculty of Medicine, Ain Shams University, Cairo, Egypt; 5 Thumbay Research Institute for Precision Medicine, Gulf Medical University, Ajman, United Arab Emirates; 6 INSERM UMR 1186, Integrative Tumor Immunology and Cancer Immunotherapy, Gustave Roussy, University Paris-Saclay, Villejuif, France

**Keywords:** cancer stem cells, hypoxia, immune evasion, immunotherapy resistance, natural killer cells, PD-L1, tumor microenvironment, tumor-associated macrophages

## Abstract

**Background:**

Hypoxia, cancer stem cell (CSC) plasticity, and immune suppression are established features of the tumor microenvironment, but their integration within the same biological systems has not been comprehensively synthesized. This systematic review evaluated whether hypoxia or hypoxia-associated signaling coordinates CSC or stemness phenotypes with immune-related mechanisms.

**Methods:**

PubMed, Embase, Scopus, Web of Science, and Google Scholar were searched for English-language studies published from 1 January 2010 to 31 January 2026. Eligible studies were required to evaluate hypoxia or hypoxia-associated signaling, CSC or stemness phenotypes, and immune-related outcomes within the same experimental or translational study. Of 1,569 records identified, 39 reports underwent full-text assessment and 14 studies were included.

**Results:**

Hypoxia-associated conditions were recurrently linked to CSC enrichment, self-renewal, epithelial-to-mesenchymal transition, tumorigenicity, metastatic potential, immune suppression, and therapeutic resistance. Four context-dependent patterns emerged: impaired natural killer, cytokine-induced killer, and CD8-positive T-cell function; myeloid- and macrophage-mediated reinforcement of CSC states through polarization, cytokine signaling, extracellular vesicles, cellular transfer, and stromal or vascular remodeling; checkpoint-associated and adaptive immune suppression involving PD-L1, CD47, regulatory T cells, and myeloid-derived suppressor cells; and an integrated hypoxic CSC–immune niche phenotype. Preclinical intervention studies indicated that selected pathways may be therapeutically modifiable, although the evidence remained predominantly experimental.

**Conclusions:**

Hypoxia appears to act as a coordinating microenvironmental pressure linking CSC plasticity with immune dysfunction in selected tumor contexts. The proposed hypoxic CSC–immune niche is a mechanistic framework rather than evidence of a universal pathway and requires validation using spatially resolved human tumors, patient-derived immune models, and treatment-response-linked cohorts.

**Systematic review registration:**

https://www.crd.york.ac.uk/PROSPERO/view/CRD420261287704, identifier CRD420261287704.

## 1 Introduction

Hypoxia is one of the defining characteristics of the tumor microenvironment (TME) in solid malignancies and develops when rapid tumor growth exceeds the capacity of the existing vasculature to supply adequate oxygen. Rather than functioning merely as a passive metabolic limitation, hypoxia initiates a broad range of adaptive transcriptional responses, primarily through activation of hypoxia-inducible factors (HIFs), particularly HIF-1α and HIF-2α (1–3). These transcriptional programs regulate numerous biological processes, including angiogenesis, metabolic reprogramming, epithelial-to-mesenchymal transition, invasion, immune evasion, and therapeutic resistance (2, 4). Consequently, hypoxia has emerged as a central driver of tumor progression and adaptation within the hostile tumor microenvironment.

Among the most important consequences of tumor hypoxia is the maintenance and expansion of cancer stem cells (CSCs). CSCs represent a specialized subpopulation of tumor cells characterized by enhanced self-renewal, tumor-initiating capacity, metastatic potential, and intrinsic resistance to conventional therapies (5, 6). Hypoxic niches provide a favorable microenvironment for CSC maintenance by regulating stemness-associated transcription factors and signaling pathways, including NANOG, SOX2, OCT4, CD44, CD133, aldehyde dehydrogenase (ALDH) activity, Wnt/β-catenin signaling, and sphere-forming capacity (7, 8). In addition to preserving pre-existing CSC populations, hypoxia may induce stem-like characteristics in differentiated tumor cells through cellular plasticity. These adaptive mechanisms contribute to tumor heterogeneity, therapeutic resistance, metastatic dissemination, and disease recurrence (9, 10).

Beyond promoting stemness, hypoxia profoundly influences the antitumor immune response. Hypoxia-associated signaling impairs immune surveillance through multiple mechanisms, including reduced natural killer (NK)-cell recognition, suppression of CD8^+^ cytotoxic T-cell function, altered immune-cell adhesion, and increased expression of immune checkpoint molecules (11, 12). Simultaneously, hypoxia promotes the recruitment and functional polarization of immunosuppressive cell populations, including regulatory T cells (Tregs), tumor-associated macrophages (TAMs), myeloid-derived suppressor cells (MDSCs), and other suppressive myeloid compartments (13, 14). Collectively, these alterations generate an immunosuppressive tumor microenvironment that favors tumor survival and disease progression.

Importantly, accumulating evidence suggests that hypoxia-mediated regulation of CSCs and immune suppression are not independent phenomena but are closely interconnected. Hypoxic tumor regions may simultaneously promote CSC maintenance while suppressing antitumor immunity, thereby creating specialized microenvironmental niches that are stemness-permissive, immune-refractory, and highly resistant to therapy. Within these niches, CSCs may further reinforce immune evasion through immune checkpoint upregulation, cytokine-mediated immunosuppression, metabolic competition, and reciprocal interactions with macrophages and other myeloid cells (14, 15). This reciprocal relationship suggests that hypoxia may function as a central coordinator linking CSC biology with immune escape mechanisms.

The convergence of hypoxia, CSC plasticity, and immune suppression has particularly important implications for cancer immunotherapy. The efficacy of immune checkpoint inhibitors and other immunotherapeutic approaches depends on efficient antigen presentation, adequate immune-cell infiltration, sustained cytotoxic activity, and the absence of dominant immunosuppressive signals within the TME. However, hypoxic, CSC-enriched tumor regions frequently exhibit resistance to immune-mediated elimination because of impaired NK- and T-cell function, increased checkpoint expression, macrophage- and myeloid-mediated CSC support, cytokine-driven immunosuppression, and metabolic dysfunction (14, 15). Understanding how hypoxia orchestrates CSC–immune interactions may therefore help explain why many tumors remain refractory to immunotherapy despite the presence of therapeutically targetable immune pathways.

Although hypoxia, CSC biology, and immune evasion have each been extensively investigated, they have largely been reviewed as separate or only partially overlapping processes. This fragmented perspective limits our understanding of whether hypoxia-induced stemness and immune suppression represent parallel consequences of tumor stress or components of a coordinated adaptive program within the tumor microenvironment. Studies that simultaneously evaluate hypoxia-associated signaling, CSC phenotypes, and immune-related mechanisms within the same experimental system are particularly valuable because they provide direct evidence of mechanistic interactions rather than simple co-occurrence.

Therefore, this systematic review synthesizes evidence from studies that concurrently investigated hypoxia or hypoxia-associated signaling, CSC or stemness phenotypes, and immune-related mechanisms within the same experimental framework. The review aims to identify recurrent molecular and cellular pathways linking hypoxia-associated CSC plasticity with immune modulation, critically evaluate the strength of evidence across heterogeneous experimental models, and assess the translational implications of these interactions for immune escape, immunotherapy resistance, and the development of tumor microenvironment-targeted therapeutic strategies.

## 2 Methods

### 2.1 Registration and reporting

This systematic review was conducted in accordance with the Preferred Reporting Items for Systematic Reviews and Meta-Analyses guidelines (16) and prospectively registered in the International Prospective Register of Systematic Reviews (PROSPERO; CRD420261287704). The review was designed to synthesize mechanistic and translational evidence from studies that concurrently assessed hypoxia or hypoxia-associated signaling, CSC or stemness phenotypes, and immune-related mechanisms within the TME.

### 2.2 Review question and PICOS framework

The review question was structured using an adapted PICOS framework suitable for mechanistic evidence synthesis. The population comprised experimental and translational cancer systems, including *in vitro* and ex vivo models, animal tumor models, human tumor tissue, serum extracellular vesicles, and public datasets supported by experimental validation. The exposure or intervention was hypoxia, hypoxia-associated signaling, or a clearly defined hypoxia-responsive system. Comparators included normoxic, untreated, genetic, pharmacologic, or other relevant controls where available; a single comparator was not mandatory because of the mechanistic scope of the review. Required outcomes included both CSC or stemness-related phenotypes and immune-related molecular, cellular, tissue-level, or functional outcomes. Eligible study designs included *in vitro*, ex vivo, animal, human tissue, dataset-supported, interventional, and mixed-design studies.

### 2.3 Search strategy

A comprehensive systematic literature search was conducted in PubMed, Embase, Scopus, Web of Science, and Google Scholar to identify relevant studies published in English between 1 January 2010 and 31 January 2026. The search strategy combined three core concepts: (i) hypoxia or hypoxia-associated signaling, (ii) CSCs or stemness-related phenotypes, and (iii) immune modulation, immune suppression, or immune escape within the TME.

For Google Scholar, the first 600 relevance-ranked records were recorded before duplicate removal and screened using the same two-team review process applied to the complete record set. Reference lists of relevant articles were also examined to identify potentially eligible reports, which were assessed against the same eligibility criteria. The complete database-specific search strategies, including search terms, Boolean operators, search dates, and applied limits, are provided in **Supplementary Data Sheet 1**.

### 2.4 Eligibility criteria

Studies were considered eligible if they concurrently evaluated all three predefined domains within the same study: (i) hypoxia or hypoxia-associated signaling, (ii) cancer stem cell (CSC) or stemness-related phenotypes, and (iii) immune-related molecular, cellular, tissue-level, or functional outcomes relevant to immune modulation. Eligible evidence of hypoxia included low-oxygen culture conditions, chemical stabilization of hypoxia-inducible signaling, activation of hypoxia-inducible factor (HIF)-1α or HIF-2α, hypoxia-associated gene expression, tumor hypoxia assessment, intermittent hypoxia, or other clearly defined hypoxia-responsive experimental systems. Eligible evidence of CSC or stemness phenotypes included the expression of CSC markers, stemness-associated transcription factors, sphere-forming ability, self-renewal capacity, tumor-initiating potential, lineage plasticity, metastatic CSC phenotypes, or the acquisition of stem-like characteristics.

Eligible immune-related outcomes included immune-cell infiltration or function, immune-mediated cytotoxicity, cytokine signaling, macrophage polarization, myeloid-derived suppressor cell (MDSC)-mediated immunosuppression, Treg recruitment, immune checkpoint regulation, immune escape mechanisms, or suppression of innate and adaptive immune responses. Experimental *in vitro*, ex vivo, animal, translational human tissue, public dataset-supported, and mixed-design studies were eligible for inclusion. Interventional or therapeutic perturbation studies were included only if they directly evaluated all three predefined domains and provided mechanistic or translational evidence linking hypoxia-associated conditions with CSC or stemness phenotypes and immune-related outcomes.

Studies were excluded if they were reviews, systematic reviews, meta-analyses, editorials, commentaries, study protocols, conference abstracts lacking sufficient primary data, non-cancer studies, case reports, or small case series. Studies that evaluated only one or two of the three required domains were also excluded. In addition, purely computational or dataset-only studies were excluded unless their findings were supported by experimental, animal, or human tissue validation.

### 2.5 Study selection and data extraction

All retrieved records were imported into Zotero for reference management. Duplicate records were identified and manually verified before removal, after which the remaining records were exported to Rayyan for screening. Title and abstract screening was conducted through two parallel reviewer teams: Prasanna Appiya Premvignesh (PAP) and Mohammed Nayeem Shaji (MNS), and Abubakker Siddiq Mohamed Hameed (ASMH) and Ibrahim Mustufa Topiwala (IMT). Each team collectively assessed the complete set of 1,153 records, with the workload divided between its two members; consequently, every record received one assessment from each reviewer team. Team-level decisions were compared, and disagreements were resolved through discussion and consensus, with consultation of a senior author when required. The same two-team approach was used for full-text assessment of all 39 reports. Within each team, the reports were divided between the two reviewers, while both teams collectively covered the complete set; each report therefore received two separate eligibility assessments overall. Discrepant inclusion or exclusion decisions were discussed, and unresolved disagreements were referred to senior authors for adjudication. Eligibility at full text required concurrent evaluation of hypoxia or hypoxia-associated signaling, CSC or stemness phenotypes, and immune-related outcomes within the same experimental or translational study. Report-specific reasons for excluding the 25 full-text reports are provided in **Supplementary Data Sheet 4**. Data extraction was independently performed by two reviewers (PAP and ASMH) for all 14 included studies using a standardized extraction framework. Extracted variables included study characteristics, country, cancer type, study model, hypoxia exposure or mechanism, CSC or stemness evidence, immune components or outcomes, proposed CSC–immune mechanisms, principal findings, and translational relevance. The independently completed extraction records were compared, and discrepancies were resolved through discussion and consensus; senior authors were consulted when clarification was required.

### 2.6 Methodological quality appraisal

Given the methodological heterogeneity of the included studies, methodological appraisal was conducted separately for each applicable evidence component rather than by applying a single overall study-level score. Appraisal was independently performed by two reviewers (PAP and ASMH) for all included studies, their domain-level judgments were compared and reconciled through discussion, and the consolidated tables were reviewed by the senior authors for methodological and scientific consistency. Animal experimental components were appraised using domains informed by the Systematic Review Centre for Laboratory Animal Experimentation (SYRCLE) Risk of Bias Tool (17). Human tissue, serum extracellular vesicle, and public dataset-supported observational components were evaluated using Newcastle–Ottawa-style domains adapted to the available evidence (18). *In vitro* and ex vivo mechanistic components were assessed using predefined domains addressing clarity of hypoxia exposure or assessment, adequacy of CSC or stemness characterization, validity of immune-related assays, appropriateness of controls or comparators, and depth of mechanistic validation. No numerical scoring, cross-domain aggregation, or combined overall rating was performed. Detailed domain-level assessments are presented in **Supplementary Tables S1**–**S3**, and **Table 1** provides a descriptive summary of the component-specific approaches and principal limitations.

**Table 1:** Descriptive summary of component-specific methodological appraisal and principal limitations.

Study	Evidence components assessed	Component-specific appraisal domains	Principal component-specific considerations
Wang et al. (20)	*In vitro* TNBC models, human tissue, NK-cell assays, and xenograft models	SYRCLE-informed animal domains; observational human-tissue domains; adapted mechanistic domains	Animal randomization, allocation concealment, blinding, and sample-size justification were incompletely reported; human-tissue inference remained observational despite strong functional NK-cell assays.
Geng et al. (21)	*In vitro* colorectal CSC and NK-cell co-culture	Adapted mechanistic domains	Functional NK-cell assays were informative, but evidence relied mainly on one colorectal cancer cell-line model without animal or human-tumor validation.
Dong et al. (24)	Human glioma samples, public datasets, macrophage assays, and orthotopic xenograft	SYRCLE-informed animal domains; observational human/dataset domains; adapted mechanistic domains	Multimodal validation was extensive, but animal bias-reduction procedures were incompletely reported and macrophage modeling relied partly on U937-derived cells.
Sami et al. (25)	TNBC cell models, animal models, and human samples	SYRCLE-informed animal domains; observational human-tissue domains; adapted mechanistic domains	Multiple experimental platforms supported the mechanism, but randomization and blinding were incompletely reported and the biology centered on an IRIS-overexpression context.
Hasmim et al. (30)	Melanoma cell model and mouse tumor model	SYRCLE-informed animal domains; adapted mechanistic domains	Direct mechanistic and *in vivo* evidence was provided, but animal bias safeguards were incompletely reported and the findings were based on a specific melanoma model.
Bu et al. (22)	Ovarian cancer spheroids and human-derived CIK cells	Adapted mechanistic domains	Human-derived CIK cells and functional cytotoxicity assays were used, but the study relied mainly on A2780 spheroids and chemically induced hypoxia without *in vivo* validation.
Liu et al. (26)	Human glioma tissues, public datasets, glioma cell model, and macrophage assays	Observational human/dataset domains; adapted mechanistic domains	The human-tissue validation set was small, and mechanistic evidence relied heavily on CoCl_2_-treated A172 cells and THP-1-derived macrophages.
Yin et al. (31)	Endometrial cancer stem-like cells and xenograft model	SYRCLE-informed animal domains; adapted mechanistic domains	Hypoxia, PD-L1, and CSC self-renewal were linked, but direct immune-effector functional testing was limited and some animal bias safeguards were incompletely described.
Hou et al. (27)	NSCLC patient tissues and serum EVs, A549 and H1299 cell models, THP-1 macrophage polarization assays, EV co-culture, and tumor-bearing mouse model	SYRCLE-informed animal domains; observational human/serum-EV domains; adapted mechanistic domains	EV signaling and rescue experiments supported the mechanism, but CSC validation was mainly marker-based and macrophage assays used THP-1 cells rather than primary TAMs.
Liu et al. (33)	4T1 and 4T1/DOX tumor models, BCSC assays, MDSC assessment, 3D tumor penetration, hypoxia-responsive HRA@D-TT system, and anti-PD-1 combination therapy	SYRCLE-informed animal domains; adapted mechanistic domains	The intervention targeted hypoxic CSC and MDSC compartments, but it was a therapeutic nanoplatform model with incomplete reporting of several animal bias safeguards.
Zhang et al. (32)	Hypoxic 4T1 assays, CSC-associated PD-L1 assessment, T-cell killing assay, BMDC maturation assay, 4T1 tumor model, immune profiling, transcriptomics, and rechallenge model	SYRCLE-informed animal domains; adapted mechanistic domains	Functional T-cell and immune-memory outcomes were assessed, but evidence arose from a treatment-reversal model with limited patient-level validation and incomplete animal bias reporting.
Jang et al. (28)	*In vitro* hypoxic and gemcitabine-stressed pancreatic cancer TME mimic, fibroblasts/CAFs, U937-derived macrophages, direct cell-to-cell transfer, CSC functional assays, multi-omic analysis, and TCGA/GEO survival analysis	Observational public-dataset domains; adapted mechanistic domains	The stressed TME mimic and CSC functional assays were informative, but immune evidence centered on macrophage-to-cancer-cell transfer rather than direct effector-cell suppression.
Fico and Santamaria-Martínez (29)	MMTV-PyMT;Tgfbi knockout mouse model, tumor sphere assays, FACS, tumor-initiation/metastasis assays, vascular and hypoxia analyses, bone marrow transplantation, human tissue, and METABRIC/CIBERSORT analysis	SYRCLE-informed animal domains; observational human/dataset domains; adapted mechanistic domains	Genetic mouse, vascular, metastatic, and human/dataset analyses were integrated, but immune findings were mainly compositional and observational rather than direct effector-cell testing.
Akbarpour et al. (23)	TC1 murine epithelial lung tumor model exposed to intermittent hypoxia or sleep fragmentation, CD8+ T-cell profiling, qPCR, histological invasion assessment, CSC marker analysis, and ex vivo CTL killing assay	SYRCLE-informed animal domains; adapted mechanistic domains	Intermittent hypoxia and CTL function were assessed *in vivo*, but CSC characterization was mainly marker-based and allocation concealment was not clearly reported.

### 2.7 Data synthesis

A meta-analysis was not performed because of substantial heterogeneity among the included studies in cancer types, experimental models, hypoxia induction or assessment methods, definitions of CSCs, immune-related endpoints, and outcome measures. Findings were therefore synthesized narratively in accordance with guidance for synthesis without meta-analysis (19). The narrative synthesis transparently described the grouping strategy, direction and consistency of findings, and limitations associated with the absence of quantitative pooling.

The synthesis focused on recurrent mechanistic patterns linking hypoxia or hypoxia-associated signaling with CSC or stemness phenotypes and immune modulation. Findings were organized into four thematic categories: (i) cytotoxic effector-cell escape, (ii) myeloid/macrophage-mediated CSC support, (iii) checkpoint-associated and adaptive immune suppression, and (iv) an integrated hypoxia-associated CSC–immune niche phenotype.

Interventional studies that therapeutically targeted hypoxic CSC–immune compartments were interpreted as providing preclinical evidence for the potential modifiability of selected pathways rather than direct evidence for the spontaneous formation of endogenous hypoxic CSC–immune niches.

A formal assessment of publication bias was not performed because of the limited number of included studies, substantial methodological heterogeneity, and the absence of pooled quantitative effect estimates.

No formal certainty-of-evidence framework, including GRADE, was applied because the review synthesized heterogeneous preclinical and translational mechanistic evidence without pooled effect estimates. Instead, the narrative synthesis described the relative breadth and coherence of support for each mechanistic pattern using the number of supporting studies, consistency of findings, depth of mechanistic validation, and component-specific methodological considerations. These comparisons were descriptive and were not treated as validated certainty categories or study-level quality grades.

## 3 Results

### 3.1 Study selection and evidence base

The systematic literature search identified 1,569 records across PubMed, Embase, Scopus, Web of Science, and Google Scholar. After removal of 416 duplicate records, 1,153 unique records underwent title and abstract screening, of which 1,114 were excluded because they did not meet the predefined eligibility criteria. The remaining 39 articles underwent full-text assessment, and 14 studies fulfilled all inclusion criteria and were included in the final qualitative synthesis (**Figure 1**).

**Figure 1:**
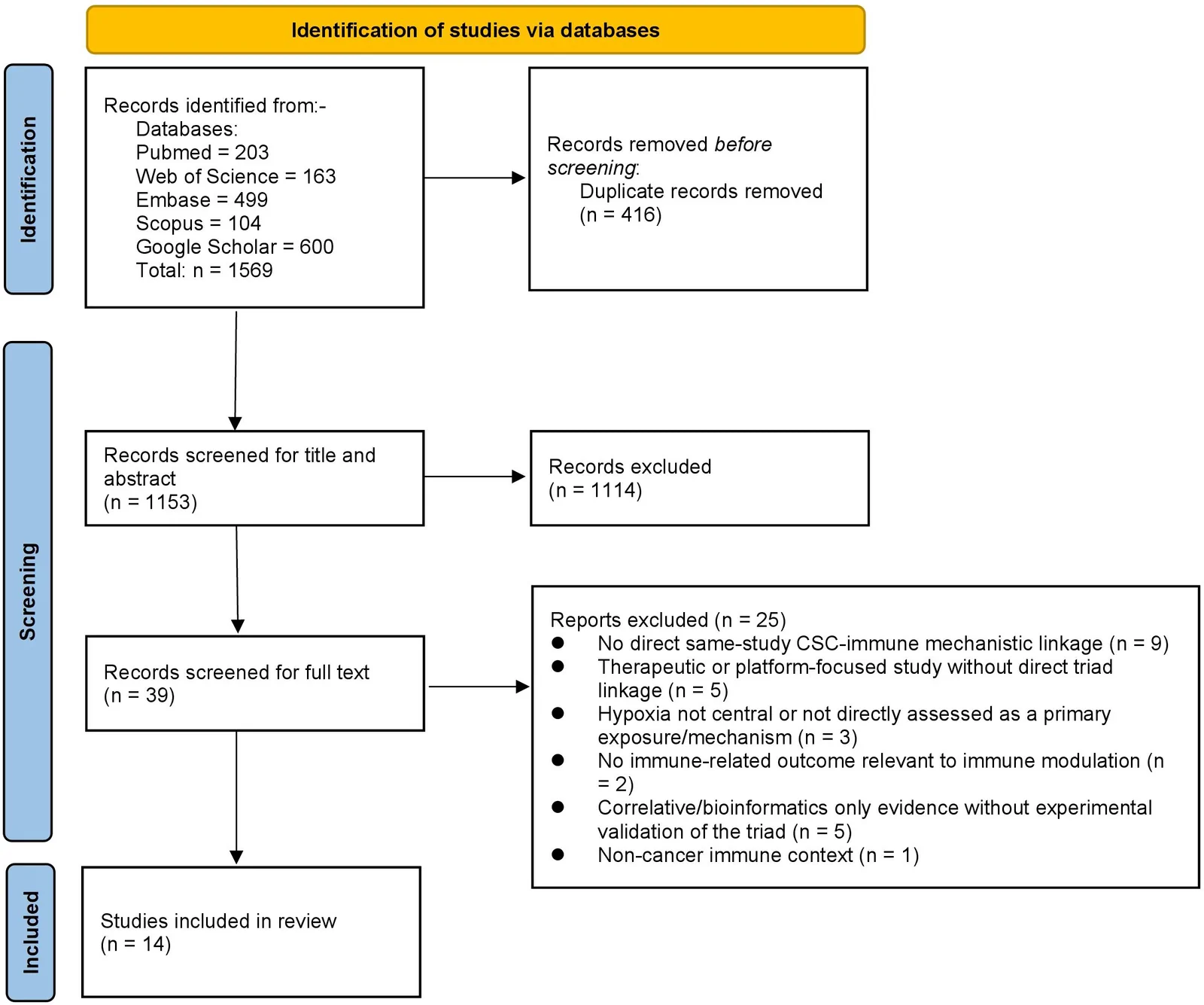
PRISMA flow diagram summarizing record identification, duplicate removal, title and abstract screening, full-text assessment, exclusion reasons, and final inclusion of 14 studies in the systematic review.

Among the 25 full-text articles excluded, the principal reasons were absence of a direct same-study mechanistic linkage between hypoxia, CSCs, and immune-related outcomes; evaluation of therapeutic platforms without assessment of the complete hypoxia–CSC–immune triad; lack of hypoxia as a primary exposure or mechanistic factor; absence of immune-related outcomes relevant to immune modulation; reliance on correlative or bioinformatics-only evidence without experimental validation of the triad; and investigation of a non-cancer immune context. All excluded full-text reports and their report-specific primary reasons for exclusion are listed in **Supplementary Data Sheet 4**.

The 14 included studies represented multiple tumor types, including triple-negative breast cancer, breast cancer, colorectal cancer, glioblastoma, melanoma, ovarian cancer, endometrial cancer, non-small cell lung cancer, pancreatic cancer, and epithelial lung tumor models. The studies used diverse designs, including *in vitro* tumor-cell models, ex vivo immune-cell assays, three-dimensional spheroids, animal studies, human tissue analyses, serum extracellular vesicle profiling, public dataset-supported investigations with experimental validation, and interventional therapeutic perturbation models.

Hypoxia was induced or assessed using controlled low-oxygen culture conditions, cobalt chloride-mediated stabilization of hypoxia-inducible signaling, intermittent hypoxia models, pimonidazole-based tumor hypoxia assessment, hypoxia-responsive therapeutic systems, and evaluation of hypoxia-inducible factor-associated signaling pathways.

Despite methodological diversity, recurrent mechanistic themes emerged. Hypoxia-associated responses were mainly mediated through HIF-1α and HIF-2α, with downstream regulation involving Wnt/β-catenin, NF-κB, transforming growth factor-β, AKT/mTOR, PD-L1, metabolic reprogramming, extracellular vesicle-mediated communication, macrophage-associated microenvironmental remodeling, vascular dysfunction, and suppression of immune effector-cell function. Collectively, these findings support a coordinating role for hypoxia in promoting CSC maintenance while shaping an immunosuppressive TME. Detailed characteristics of the included studies are presented in **Supplementary Data Sheet 2**.

### 3.2 Hypoxia linked CSC plasticity to immune suppression across recurrent mechanistic patterns

Across the included studies, hypoxia-associated conditions were recurrently associated with CSC enrichment, self-renewal, epithelial-to-mesenchymal transition, tumorigenicity, metastatic potential, therapeutic resistance, and immune suppression. Although the molecular pathways varied among tumor types and experimental systems, the evidence converged on four recurrent, context-dependent patterns linking hypoxia-associated CSC plasticity with immune regulation: (i) cytotoxic effector-cell escape, (ii) myeloid/macrophage-mediated CSC support, (iii) checkpoint-associated and adaptive immune suppression, and (iv) an integrated hypoxia-associated CSC–immune niche phenotype. Several interventional studies additionally provided preclinical evidence that selected pathways may be therapeutically targetable (**Table 2**; **Figure 2**).

**Table 2:** Mechanistic and translational synthesis of hypoxia-driven CSC–immune interactions.

Mechanistic pattern	Supporting studies	Core pathway	Immune consequence	CSC consequence
Cytotoxic effector-cell escape	Wang et al. (20); Geng et al. (21); Bu et al. (22); Akbarpour et al. (23); Zhang et al. (32)	HIF-1α/Akt/Wnt/Frizzled-7/MICA-NKG2D; FADS1/DDA axis and spermine-associated NK suppression; HIF-1α-TGF-β1/SMAD-VEGFA-ICAM-1; intermittent hypoxia-associated CD8+ T-cell dysfunction; hypoxia-relieving AMPK-mediated PD-L1 degradation in CSCs	Reduced NK recognition, reduced NK cytotoxicity, NK-cell apoptosis, reduced CIK-cell killing, impaired CD8+ cytotoxic function, reduced granzyme B, perforin, IFN-γ, and improved T-cell killing after hypoxia-targeted intervention	CSC enrichment, CSC survival, increased Oct4+ or CD44+CD133+ CSC-like populations, and reduced CSC immune escape after therapeutic hypoxia modulation
Myeloid/macrophage-mediated CSC support	Dong et al. (24); Sami et al. (25); Liu et al. (26); Hou et al. (27); Jang et al. (28); Fico and Santamaria-Martínez (29)	TREM1-TLR2/AKT/mTOR/c-MYC-TGF-β2; BRCA1-IRIS-GM-CSF/TGF-β1 axis; PGCC-TAM axis; hypoxic EV/SNHG16-miR-132-3p-KIF5A signaling; macrophage-to-cancer-cell transfer under hypoxic and chemotherapy-stressed TME conditions; TGFBI-mediated vascular dysfunction and hypoxia	M2-like TAM polarization, IL-10/TGF-β-rich suppression, macrophage-mediated CSC support, macrophage-to-tumor transfer of cellular components, myeloid/M2 enrichment, and reduced CD8+ T-cell content	GSC/CSC plasticity, EMT, mesenchymal transition, CSC-like derivative cell formation, tumor sphere/mammosphere formation, ALDHhigh CSC enrichment, metastatic CSC support, and tumor-initiating capacity
Checkpoint-associated and adaptive immune suppression	Sami et al. (25); Hasmim et al. (30); Yin et al. (31); Zhang et al. (32); Liu et al. (33)	BRCA1-IRIS/HIF-1α/NF-κB-PD-L1/CD47; NANOG-TGF-β1; HIF-1α/HIF-2α-PD-L1; AMPK-mediated membrane PD-L1 degradation on tumor cells and CSCs; hypoxia-responsive ATRA release targeting CSC and MDSC compartments	Treg recruitment, CD8+ T-cell suppression, checkpoint-mediated immune escape, PD-L1-associated immune evasion, reduced MDSC-mediated immunosuppression, increased DC maturation, increased CD8+ T-cell infiltration, and improved anti-PD-1 response after intervention	CSC self-renewal, tumorigenicity, CSC-associated PD-L1 expression, reduced CSC populations after hypoxia-targeted intervention, and differentiation of CSC-like compartments
Cross-cutting hypoxic niche phenotype	Wang et al. (20); Dong et al. (24); Hasmim et al. (30); Liu et al. (26); Yin et al. (31); Jang et al. (28); Fico and Santamaria-Martínez (29); Akbarpour et al. (23)	HIF-1α/HIF-2α with Wnt/β-catenin, TGF-β, NANOG, PD-L1, hypoxia-stress TME remodeling, vascular dysfunction, intermittent hypoxia, and stemness-associated hypoxic signaling	Immune-refractory niche formation involving impaired cytotoxic recognition, macrophage/myeloid support, reduced CD8+ T-cell function, immune checkpoint activity, and spatially restricted hypoxic immune remodeling	Stemness-permissive tumor state involving CSC enrichment, lineage plasticity, metabolic adaptation, tumorigenicity, metastasis, and therapy-resistant hypoxic niche behavior
Interventional/translational targeting of hypoxic CSC-immune niches	Liu et al. (33); Zhang et al. (32)	Hypoxia-responsive HRA@D-TT-mediated ATRA delivery to deep tumor regions; CuET/ICG-mediated mitochondrial interference, reduced oxygen consumption, hypoxia relief, PDT-amplified immunogenic cell death, and AMPK-mediated PD-L1 degradation	Reduced MDSC-associated immunosuppression, improved anti-PD-1 response, increased DC maturation, increased CD8+ T-cell infiltration, reduced Tregs, enhanced T-cell killing, and induction of immune memory	Differentiation or reduction of CSC-like populations, reduced CSC-associated PD-L1, decreased stemness-associated pathways, and improved vulnerability of CSC-containing tumors to chemoimmunotherapy

**Figure 2:**
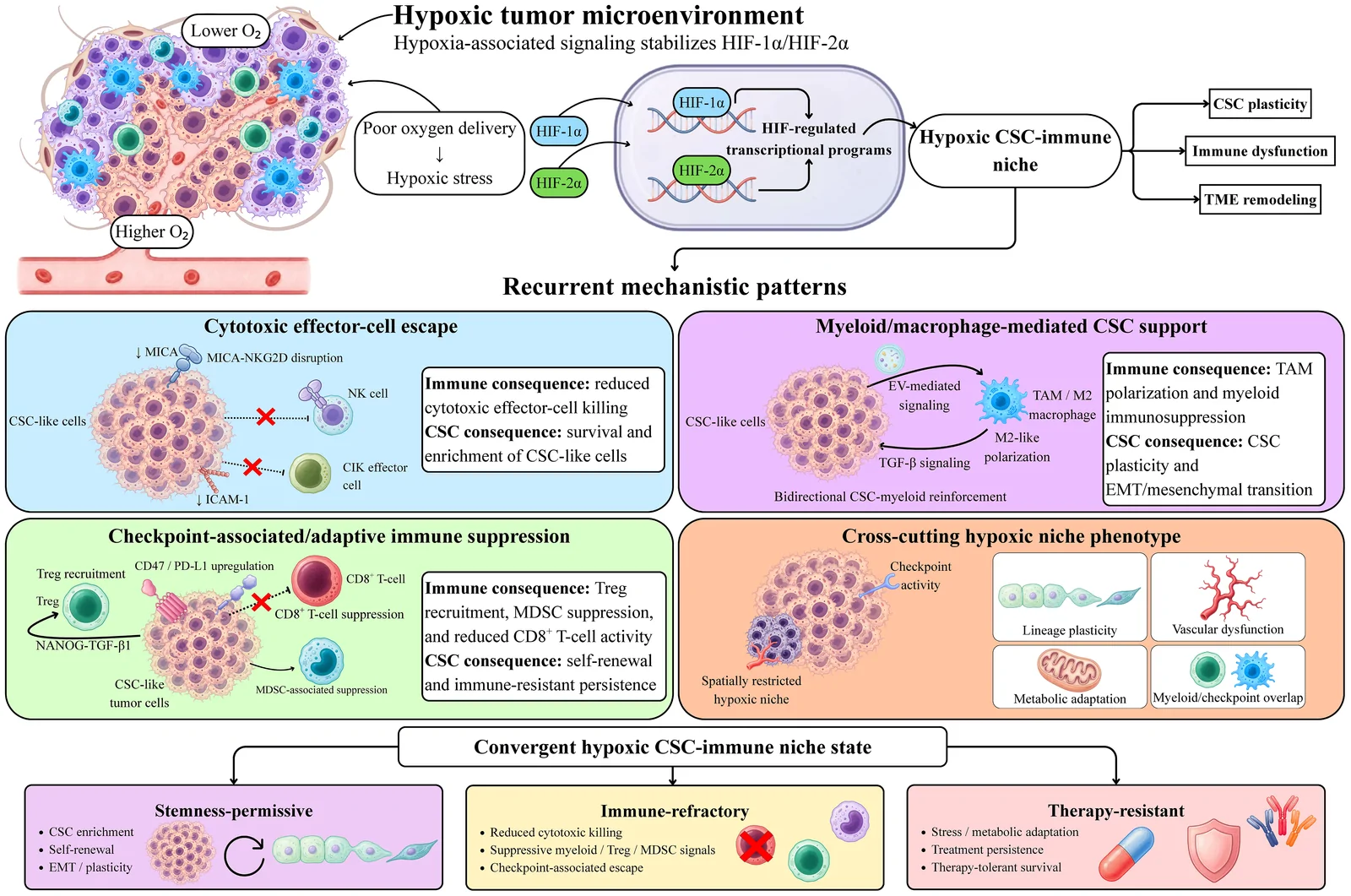
Mechanistic model of hypoxic cancer stem cell–immune niches within the tumor microenvironment.

#### 3.2.1 Cytotoxic effector-cell escape

The first recurring pattern involved impaired recognition and elimination of hypoxic CSC-like tumor cells by cytotoxic immune effectors, particularly NK cells, CIK cells, and CD8^+^ cytotoxic T lymphocytes. In triple-negative breast cancer (TNBC), hypoxia-induced HIF-1α activation stimulated the Akt/Wnt/β-catenin pathway and increased Frizzled-7 expression, resulting in enrichment of CD44^+^/CD24^-^ and ALDH1^+^ CSC populations. These changes were accompanied by impaired MICA–NKG2D-mediated NK-cell recognition, reduced NK-cell infiltration and activation, and decreased expression of CD107a, granzyme B, and perforin (20). In colorectal CSC–NK-cell co-culture models, hypoxia-associated metabolic remodeling involving the FADS1/DDA axis and increased spermine levels was associated with reduced NK-cell cytokine secretion, impaired cytotoxicity, and increased NK-cell apoptosis (21). In ovarian cancer spheroids, hypoxia-associated HIF-1α stabilization activated TGF-β1/SMAD and VEGFA signaling, resulting in ICAM-1 downregulation and reduced CIK-cell adhesion and tumor-cell lysis (22). Likewise, in a TC1 murine epithelial lung tumor model, intermittent hypoxia reduced granzyme B-producing intratumoral CD8^+^ T cells, decreased granzyme B, perforin, and IFN-γ expression, impaired cytotoxic T-lymphocyte-mediated tumor killing, and increased Oct4^+^ and CD44^+^/CD133^+^ CSC-like populations (23).

Collectively, these findings indicate that hypoxia-associated CSC-like states may facilitate immune escape through impaired immune recognition, defective immune-cell adhesion, metabolic suppression, and reduced cytotoxic effector function.

#### 3.2.2 Myeloid- and macrophage-mediated reinforcement of CSC phenotypes

The second recurring pattern involved reciprocal interactions between hypoxic tumor cells and the myeloid compartment, particularly tumor-associated macrophages (TAMs), which reinforced CSC plasticity and established an immunosuppressive tumor microenvironment. In glioblastoma, hypoxia-induced HIF-1α signaling activated TREM1–TLR2/AKT/mTOR/c-MYC signaling and increased TGF-β2 secretion, promoting TGF-β receptor/SMAD activation in glioma stem cells and driving a mesenchymal-like transition (24). In BRCA1-IRIS-overexpressing TNBC models, hypoxia-mediated HIF-1α/NF-κB activation stimulated GM-CSF secretion and TAM polarization, establishing a TGF-β1-dependent feedback loop that promoted CSC and EMT phenotypes, increased regulatory T-cell (Treg) infiltration, suppressed CD8^+^ T-cell activity, and upregulated the immune-evasion molecules PD-L1 and CD47 (25).

Additional evidence further supported this mechanism. In glioblastoma, hypoxia-induced polyploid giant cancer cells and their stem-like progeny promoted polarization of CD163^+^/IL-10^+^ M2-like macrophages (26). In non-small cell lung cancer (NSCLC), hypoxic tumor-derived extracellular vesicles enriched with SNHG16 enhanced CSC-like properties and M2 macrophage polarization through the SNHG16/miR-132-3p/KIF5A signaling axis (27). In pancreatic cancer, hypoxic and gemcitabine-stressed tumor microenvironment-mimicking conditions facilitated direct macrophage-to-cancer-cell transfer, generating CD44^high/SLC16A1^high tumor cells with enhanced stemness, invasiveness, and metabolic reprogramming (28). Likewise, macrophage- and stromal-derived TGFBI promoted vascular dysfunction, tumor hypoxia, CSC enrichment, metastatic potential, M2 macrophage accumulation, and depletion of CD8^+^ T cells in breast cancer (29).

Among the identified patterns, myeloid- and macrophage-mediated remodeling provided comparatively strong evidence for reciprocal interactions between hypoxia, CSC plasticity, and immune suppression, highlighting the role of the myeloid compartment in maintaining selected hypoxia-associated CSC niches.

#### 3.2.3 Checkpoint-associated and adaptive immune suppression

The third recurring pattern involved hypoxia-mediated activation of adaptive immune suppression through immune checkpoint regulation and immunosuppressive signaling pathways. In melanoma, hypoxia-induced HIF-1α stimulated NANOG expression, which directly regulated TGF-β1, promoting Treg expansion, macrophage-mediated immune suppression, reduced CD8^+^ T-cell activity, and increased arginase-1 activity (30). In endometrial cancer, both HIF-1α and HIF-2α enhanced PD-L1 transcription while simultaneously promoting CSC self-renewal, sphere formation, colony formation, proliferation, and tumorigenicity (31). Although immune effector-cell assays were limited in this study, the findings support a mechanistic association between hypoxia-driven checkpoint signaling and maintenance of stem-like tumor states.

Similarly, the BRCA1-IRIS-overexpressing TNBC model demonstrated coordinated activation of HIF-1α/NF-κB, increased PD-L1/CD47 expression, enhanced Treg infiltration, and suppression of CD8^+^ T-cell responses (25). Complementary evidence was provided by an interventional TNBC model in which CuET/ICG nanoparticles alleviated tumor hypoxia, activated AMPK-mediated degradation of membrane PD-L1, enhanced dendritic cell maturation, increased CD8^+^ T-cell infiltration, reduced Treg abundance, and improved T-cell-mediated tumor killing (32). Overall, these findings indicate that hypoxia links CSC plasticity with adaptive immune suppression by coordinating immune checkpoint activation, immunosuppressive cytokine signaling, and inhibition of cytotoxic T-cell function.

#### 3.2.4 An integrated hypoxic CSC–immune niche

The fourth recurring pattern represented an integrated hypoxia-associated niche phenotype rather than a single signaling pathway. Across multiple tumor models, hypoxia-associated conditions were accompanied by combinations of HIF activation, CSC enrichment, lineage plasticity, epithelial-to-mesenchymal transition, macrophage or myeloid remodeling, impaired cytotoxic immunity, immune checkpoint activation, vascular dysfunction, metabolic adaptation, and therapy resistance.

Evidence supporting this integrated phenotype was observed across TNBC, glioblastoma, melanoma, endometrial cancer, pancreatic cancer, breast cancer, and epithelial lung tumor models (20, 23, 24, 26, 28–31). These convergent findings support a proposed niche framework in which hypoxia-associated CSC plasticity and immune suppression interact within selected experimental systems; however, direct spatial organization of these components within human tumors was rarely demonstrated.

### 3.3 Translational evidence for therapeutic targeting of hypoxic CSC–immune niches

Two studies provided preclinical interventional evidence that selected hypoxic CSC–immune pathways may be therapeutically targetable. Liu et al. (33) developed a hypoxia-responsive HRA@D-TT delivery system that released all-trans retinoic acid within deeply hypoxic tumor regions, promoted differentiation of CSCs and myeloid-derived suppressor cells, reduced chemoresistance and immunosuppression, and enhanced anti-PD-1-based chemoimmunotherapy. Zhang et al. (32) used CuET/ICG nanoparticles combined with photodynamic therapy to reduce oxygen consumption, alleviate tumor hypoxia, induce immunogenic cell death, promote AMPK-dependent degradation of membrane PD-L1 on tumor cells and CSCs, and enhance antitumor immune activation. Because both studies relied on therapeutic perturbation models, they were interpreted as evidence of potential pathway modifiability rather than direct proof of spontaneous endogenous niche formation.

### 3.4 Translational maturity and component-specific methodological considerations

The translational maturity of the included evidence varied considerably across studies, reflecting differences in experimental design, validation strategies, and model complexity. Studies integrating mechanistic experiments with functional immune assays, animal models, human tissue analyses, serum extracellular vesicle profiling, public dataset-supported validation, or clinically relevant therapeutic-response models provided broader triangulation than single-platform studies. Wang et al. (20), Dong et al. (24), Sami et al. (25), Hou et al. (27), and Fico and Santamaria-Martínez (29) combined tumor-cell experiments with one or more complementary validation platforms. Akbarpour et al. (23) provided disease-relevant *in vivo* evidence linking intermittent hypoxia with impaired CD8^+^ T-cell cytotoxicity and expansion of CSC-associated populations, while Jang et al. (28) combined a pancreatic cancer TME-mimicking model with public dataset support to examine macrophage-to-cancer-cell transfer under hypoxic and chemotherapy-associated stress.

The most direct preclinical interventional evidence was provided by Liu et al. (33) and Zhang et al. (32), both of which perturbed hypoxic CSC–immune compartments in therapeutic models. Modulation of tumor hypoxia was associated with CSC differentiation or depletion, enhanced antitumor immune activation, and improved immunotherapy-related outcomes in experimental systems. These studies support the potential modifiability of selected pathways but do not establish spontaneous endogenous niche formation or clinical efficacy.

Several studies provided more focused mechanistic evidence with narrower validation. Geng et al. (21) and Bu et al. (22) used *in vitro* or ex vivo immune-cell systems to investigate hypoxia-associated dysfunction of NK cells and CIK cells, respectively, but lacked extensive animal or human-tumor validation. Yin et al. (31) linked hypoxia, PD-L1 expression, and CSC self-renewal in endometrial cancer, although direct immune-effector testing was limited. Hasmim et al. (30) established a mechanistic link between hypoxia-induced NANOG expression and TGF-β1-mediated immune suppression in melanoma, but within a specific experimental context.

The component-specific appraisal showed that the evidence base was mechanistically informative but predominantly preclinical. Animal components frequently lacked detailed reporting of allocation concealment, blinding, and sample-size justification. Human tissue, serum extracellular vesicle, and public dataset-supported components improved translational relevance but remained observational, selected, or based on relatively small validation sets. *In vitro* and ex vivo components often relied on single tumor cell lines, immortalized or non-autologous immune-cell systems, chemically induced or otherwise artificial hypoxia, and CSC definitions based mainly on phenotypic markers. These considerations are reported separately by evidence component in **Table 1** and **Supplementary Tables S1**–**S3**; they were not aggregated into a single study-level rating.

Descriptively, cytotoxic effector-cell escape and myeloid/macrophage-mediated CSC support were recurrent across several experimental platforms and included functional immune assays. Checkpoint-associated and adaptive immune suppression was also represented, but the supporting studies were more heterogeneous and sometimes relied on checkpoint expression, surrogate markers, or therapeutic reversal models rather than direct endogenous immune-cell functional analyses. The integrated niche phenotype therefore reflects convergence across related observations rather than a formal certainty grade.

Taken together, the proposed hypoxic CSC–immune niche should be interpreted as a context-dependent mechanistic synthesis rather than definitive evidence of a universal or fully bidirectional pathway across cancers. Recurrence of related patterns across diverse malignancies and experimental systems nevertheless provides a rationale for studies that combine mechanistic perturbation with spatially resolved and clinically representative human validation.

## 4 Discussion

This systematic review synthesized evidence from 14 studies that concurrently evaluated hypoxia or hypoxia-associated signaling, CSC or stemness phenotypes, and immune-related mechanisms within the same experimental framework. By requiring all three domains to be investigated within individual studies, the review prioritized directly linked evidence rather than indirect inference across separate literatures. The findings converged on four recurrent and partially overlapping patterns: cytotoxic effector-cell escape, myeloid/macrophage-mediated reinforcement of CSC phenotypes, checkpoint-associated and adaptive immune suppression, and an integrated hypoxia-associated CSC–immune niche phenotype. These patterns were observed across multiple cancer types despite substantial heterogeneity in experimental models and hypoxia induction strategies, suggesting cross-context recurrence while requiring broader validation.

Among these patterns, the comparatively strongest and most consistent evidence supported cytotoxic effector-cell dysfunction and myeloid/macrophage-mediated CSC support. Multiple studies showed that hypoxia-associated conditions impaired NK-cell, CIK-cell, or CD8^+^ T-cell activity while promoting macrophage polarization, myeloid remodeling, and CSC maintenance. Evidence linking hypoxia to checkpoint-associated and adaptive immune suppression was biologically compelling but more heterogeneous, because several studies relied mainly on checkpoint expression or therapeutic perturbation rather than direct assessment of endogenous immune effector-cell function. The proposed niche should therefore be regarded as a conceptual and mechanistic framework rather than a uniform process operating identically across tumors.

Hypoxia may enhance CSC traits through both tumor-cell-intrinsic transcriptional programs and reciprocal signals from the surrounding microenvironment. Stabilization of HIF-1α or HIF-2α can promote self-renewal, survival, metabolic adaptation, and lineage plasticity. In triple-negative breast cancer, HIF-1α/Akt/Wnt/β-catenin signaling increased Frizzled-7 expression and enriched CD44^+^/CD24^-^ and ALDH1^+^ CSC-like populations (20). In melanoma, HIF-1α-dependent NANOG induction supported ALDH activity, spheroid formation, and tumorigenicity while also increasing TGF-β1-mediated immunosuppression (30). In endometrial cancer stem-like cells, HIF-1α/HIF-2α-driven PD-L1 transcription was associated with sphere formation, pluripotency-marker expression, and tumorigenicity (31). These findings indicate that HIF signaling can reinforce stemness directly through self-renewal networks and indirectly through survival and immune-evasion programs.

Hypoxia may also sustain CSC phenotypes by remodeling macrophage, myeloid, metabolic, and extracellular-vesicle signaling. In triple-negative breast cancer, HIF-1α/NF-κB-dependent GM-CSF promoted macrophage recruitment and TGF-β1-mediated CSC and epithelial-to-mesenchymal transition traits (25). In glioblastoma, HIF-1α-induced TREM1 signaling activated the TLR2/AKT/mTOR/c-MYC and TGF-β2 pathways, supporting a mesenchymal-like glioma stem-cell transition (24). Hypoxic extracellular vesicles and FADS1/DDA–spermine metabolic remodeling further linked CSC-like states to macrophage polarization or impaired NK-cell function (21, 27). Thus, hypoxia does not act through one universal pathway; instead, it reinforces CSC plasticity through context-dependent combinations of transcriptional reprogramming, metabolic adaptation, immune escape, and reciprocal stromal or myeloid support.

The immunological significance of these findings extends beyond the established role of hypoxia as a generalized metabolic stressor. The included studies suggest that immune dysfunction can arise in functional association with stemness-enriched tumor states. This is important because contemporary immunotherapies depend on cytotoxic immune cells recognizing, infiltrating, and eliminating tumor populations that contribute to persistence, metastasis, and recurrence. When hypoxia-associated CSC-like states coincide with impaired NK-cell, CIK-cell, or CD8^+^ T-cell activity, macrophage- or MDSC-mediated suppression, and checkpoint-associated immune escape, the most treatment-resistant tumor compartments may also become relatively immune refractory.

The triad-based selection strategy was a key strength of this review. Studies examining hypoxia, CSC biology, or immune suppression separately cannot establish whether these processes are linked within the same biological context. Requiring concurrent evaluation of all three domains increased the relevance of the included evidence for functional integration. However, same-study evidence does not by itself demonstrate spatial co-localization within an anatomically defined niche; direct spatial organization of hypoxia, CSC states, and immune components remains to be established in human tumors.

The recurrent hypoxia-associated CSC–immune interactions also have translational implications. Although the evidence remains predominantly preclinical, intervention studies suggested that targeting hypoxic tumor compartments may reduce CSC-like populations, attenuate MDSC- and PD-L1-associated immune escape, and improve immune-related antitumor effects in experimental models (32, 33). These observations support further investigation of combination strategies that integrate hypoxia modulation, CSC targeting, and immune restoration, while emphasizing the need for validation in clinically representative models and prospective human cohorts.

Overall, the evidence supports hypoxia as a potential coordinating microenvironmental pressure linking CSC plasticity with immune suppression through multiple context-dependent mechanisms. This framework provides a basis for future studies to define the spatial organization, molecular regulation, functional significance, and therapeutic vulnerability of hypoxia-associated CSC–immune interactions across cancer types.

### 4.1 Translational implications

The main translational implication of this review is that hypoxia, CSC plasticity, and immune suppression may need to be evaluated together rather than as isolated biomarkers. Hypoxia markers alone may identify oxygen-deprived tumor regions without determining whether those regions contain stem-like and immune-refractory cell states. CSC markers alone may identify tumor-initiating or therapy-resistant populations without defining the immune context that permits their persistence. Immune markers alone may describe cytotoxic dysfunction, macrophage polarization, or checkpoint expression without revealing whether these immune states are linked to hypoxic stemness programs.

A more informative biomarker strategy may therefore require integrated assessment of all three domains. Candidate combinations may include HIF-1α, HIF-2α, CAIX, or pimonidazole-defined hypoxia; CSC or stemness markers such as CD44, CD24, CD133, ALDH, NANOG, SOX2, OCT4, Frizzled-7, or sphere-forming capacity; and immune-contexture markers such as MICA-NKG2D signaling, granzyme B, perforin, IFN-γ, CD8-positive T-cell infiltration, Tregs, TAM or M2 macrophage markers, MDSCs, PD-L1, CD47, or cytokine profiles. These combinations remain candidate markers and require validation in spatially resolved human tumors and treatment-response-linked cohorts before clinical translation.

A translational roadmap for identifying, validating, and therapeutically targeting hypoxic CSC–immune niches is summarized in **Figure 3**. The first step in this roadmap is integrated niche identification. This requires combined assessment of hypoxia, stemness, and immune-contexture markers rather than reliance on any single marker group. For example, HIF-1α, HIF-2α, CAIX, or pimonidazole-defined hypoxia may help identify oxygen-deprived tumor regions, but these markers should ideally be interpreted together with CSC-associated markers and immune-cell or checkpoint features. The second step is functional validation. Spatial co-localization alone would not be sufficient to prove a biologically active niche. Validation should test whether hypoxic stemness-enriched compartments are associated with impaired cytotoxic activity, macrophage or myeloid support, checkpoint-associated immune evasion, or resistance to therapy (8, 14, 15).

**Figure 3:**
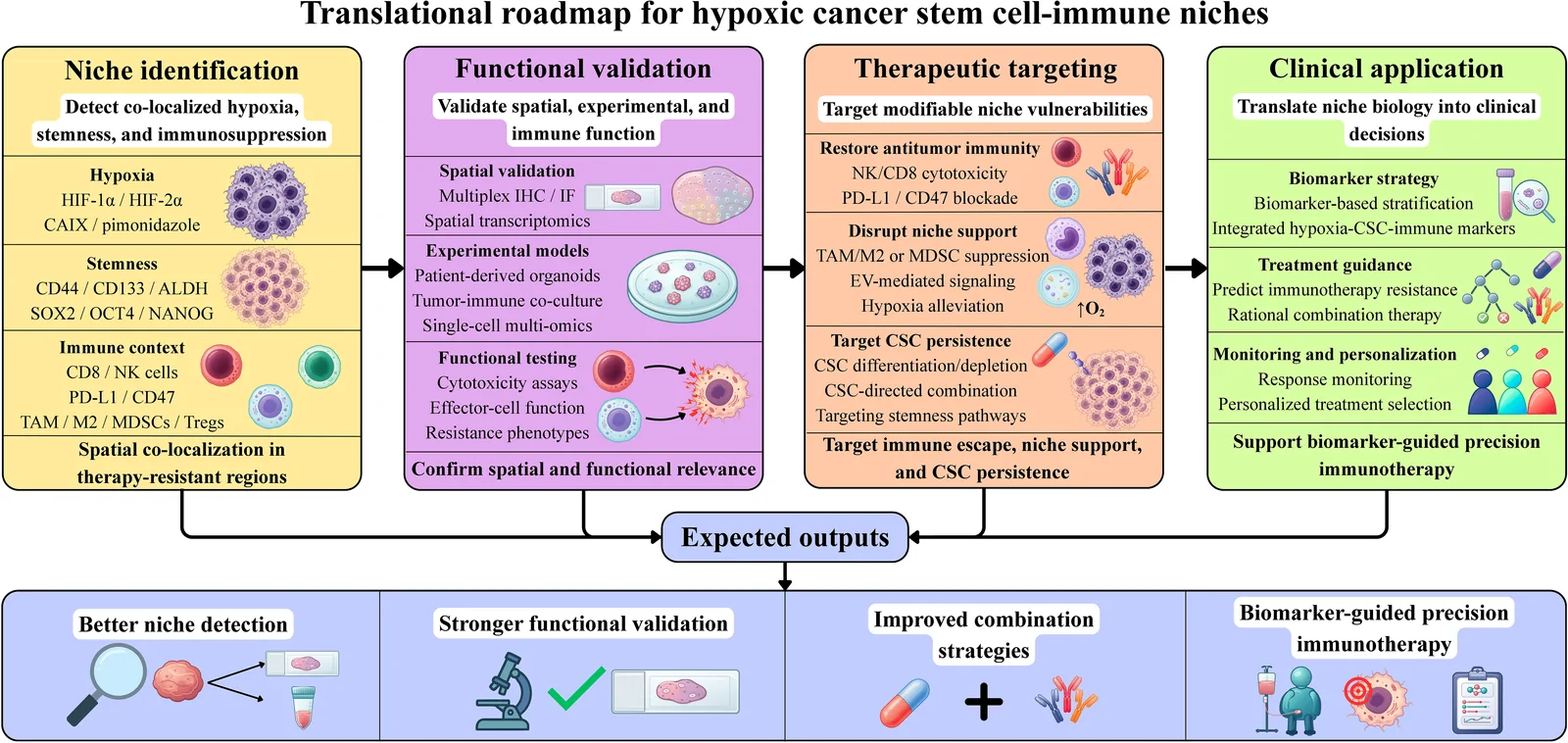
Translational roadmap for hypoxic cancer stem cell–immune niches. The image outlines a practical framework for identifying, validating, and therapeutically targeting hypoxic cancer stem cell–immune niches within the tumor microenvironment. The roadmap integrates niche identification using hypoxia, stemness, and immune-contexture markers; functional validation through spatial and experimental platforms; therapeutic targeting of niche vulnerabilities; and potential clinical applications in biomarker-based stratification, prediction of treatment resistance, rational combination therapy design, response monitoring, and personalized immunotherapy selection.

Several experimental platforms could support this validation process. Multiplex immunohistochemistry or immunofluorescence can define whether hypoxia, CSC, and immune markers occupy the same tissue regions. Spatial transcriptomics and single-cell multi-omics can help determine whether hypoxic tumor-cell states, stemness programs, and immune-suppressive compartments co-exist at cellular or regional resolution. Patient-derived organoids and autologous tumor-immune co-culture systems could then test whether these compartments are functionally resistant to immune killing and whether targeting hypoxia-associated pathways alters CSC or immune phenotypes. Treatment-response-linked cohorts are also important because the clinical relevance of these niches depends on whether they predict recurrence, metastasis, immunotherapy resistance, or failure of combined treatment strategies (14, 15).

The included studies suggest several potential therapeutic vulnerabilities, including restoration of NK-cell or CD8^+^ T-cell cytotoxicity, reversal of hypoxia-associated metabolic immune suppression, disruption of macrophage-mediated CSC support, inhibition of extracellular vesicle-mediated tumor–macrophage signaling, modulation of vascular hypoxia, targeting of PD-L1- or CD47-associated immune escape, and differentiation or depletion of CSC and MDSC compartments. Liu et al. (33) reported that hypoxia-responsive delivery of all-trans retinoic acid into deep tumor regions reduced CSC and MDSC compartments and improved anti-PD-1-based chemoimmunotherapy in experimental models. Zhang et al. (32) reported that hypoxia-relieving mitochondrial interference reduced CSC-associated PD-L1 and enhanced T-cell-mediated antitumor immunity. These findings support the possibility that selected hypoxia-associated CSC–immune pathways may be preclinically targetable, while remaining distinct from direct evidence of endogenous niche formation.

These findings suggest that therapeutic development should not be framed simply as broad inhibition of hypoxia. A more realistic strategy may involve rational combinations that target different components of the hypoxic CSC–immune niche. For example, hypoxia-responsive delivery systems may improve drug penetration into oxygen-deprived tumor regions, while CSC-directed differentiation or depletion strategies may reduce the stem-like compartment that supports relapse and resistance. At the same time, immune-restorative approaches may be needed to improve NK-cell or CD8-positive T-cell cytotoxicity, reduce MDSC- or TAM-associated suppression, or disrupt checkpoint-associated immune escape. In this model, hypoxia modulation, CSC targeting, and immune restoration are complementary rather than competing therapeutic directions (14, 34, 35).

The two interventional studies included in this review support this concept but should be interpreted cautiously. Liu et al. (33) and Zhang et al. (32) suggest that targeting hypoxic CSC–immune compartments can improve immune-related antitumor effects in experimental systems. However, these findings do not yet establish which patients would benefit, which biomarkers should guide treatment selection, or whether similar effects occur in naturally evolving human tumor niches. Future therapeutic studies should therefore integrate biomarker-defined niche detection with functional immune readouts and treatment-response endpoints (15, 32, 33). This would help determine whether hypoxic CSC–immune niches are only mechanistically interesting or whether they can become actionable targets for combination immunotherapy.

The immediate clinical implication is therefore not routine use of a specific hypoxia-targeting agent, but improved design of translational and early-phase trials. Such studies could prospectively stratify patients using integrated hypoxia–CSC–immune biomarkers and collect paired tumor biopsies before treatment and during treatment. Comparing these samples could show whether an intervention reaches the intended hypoxic compartment and whether it changes CSC burden, cytotoxic immune function, myeloid composition, checkpoint activity, and treatment response. Combination regimens should be selected according to the dominant biological feature of the niche. Examples include hypoxia modulation or hypoxia-responsive delivery combined with CSC differentiation or depletion and with immune checkpoint blockade or restoration of cytotoxic-effector function. Because HIF signaling contributes to normal physiological adaptation to low oxygen and can exert context-dependent effects in tumor and immune cells, systemic HIF inhibition requires careful safety monitoring and biomarker evidence of target engagement before clinical benefit can be inferred (14, 15, 34, 35).

Clinical translation also remains challenging because CSC markers are not uniform across cancer types, immune endpoints vary widely, and some models rely on artificial hypoxia induction or non-autologous immune systems. Future therapeutic strategies may therefore need biomarker-guided and context-specific targeting of hypoxic CSC–immune niches rather than nonspecific inhibition of hypoxia pathways.

### 4.2 Strengths, limitations, and future directions

A major strength of this review is its triad-based eligibility strategy. By requiring concurrent evaluation of hypoxia or hypoxia-associated signaling, CSC/stemness phenotypes, and immune-related mechanisms within the same study, the review reduced indirect extrapolation and focused on integrated experimental evidence. This approach allowed identification of recurrent mechanistic patterns that would be less visible in reviews addressing hypoxia, CSC biology, or immune evasion separately.

The review also benefits from methodological breadth. The included studies incorporated *in vitro* tumor models, immune-cell co-culture systems, spheroids, animal models, human tissue, serum extracellular vesicles, public datasets, vascular and hypoxia assessment, macrophage-transfer systems, intermittent hypoxia models, and immunotherapy-oriented interventional systems. This diversity allowed synthesis across several tumor contexts and helped identify recurring relationships between hypoxia, CSC plasticity, and immune regulation.

Several limitations should be considered. First, only 14 studies met the strict triad-based eligibility criteria, limiting the volume of evidence available for each mechanistic pattern and cancer type. The evidence was predominantly preclinical; although some studies included human tissue, serum extracellular vesicles, public datasets, or patient-cohort analyses, few directly validated the complete hypoxia–CSC–immune axis in spatially resolved human tumors. The studies were also substantially heterogeneous in cancer type, hypoxia model, CSC definition, immune endpoint, and validation depth. Low-oxygen culture, cobalt chloride, intermittent hypoxia, HIF-associated signaling, hypoxia-responsive therapeutic systems, pimonidazole staining, and tissue-level hypoxia assessments are not interchangeable and may represent different biological states.

CSC identification also varied substantially. Some studies used functional assays such as sphere formation, self-renewal, tumor initiation, metastatic CSC assessment, or anchorage-independent growth, whereas others relied mainly on markers including CD44, CD24, CD133, ALDH, SOX2, OCT4, NANOG, or Frizzled-7. Marker-based definitions may incompletely capture functional stemness. Immune endpoints ranged from direct NK-cell, CIK-cell, CD8^+^ T-cell, or T-cell killing assays to macrophage polarization, immune-cell composition, cytokine signaling, checkpoint expression, MDSC markers, or dataset-derived immune estimates, limiting direct comparison. Artificial hypoxia, single-cell-line systems, immortalized or non-autologous immune models, and species-specific tumor models further constrain extrapolation to naturally evolving human tumors.

Several studies used therapeutic perturbation systems, which demonstrate potential modifiability but are not equivalent to endogenous mechanistic evidence. Animal components often lacked clear reporting of allocation concealment, blinding, or sample-size justification, while human tissue and dataset-supported components remained observational and vulnerable to selection and confounding. The component-specific appraisal domains were adapted to heterogeneous evidence types and have not been validated as a unified instrument; accordingly, no numerical scoring or combined study-level rating was applied. The review was restricted to English-language studies published from 2010 onward, Google Scholar screening was limited to the first 600 relevance-ranked records, and formal publication-bias assessment was not feasible. These factors may have contributed to selection, publication, and interpretive bias.

No formal certainty-of-evidence assessment was undertaken, consistent with the heterogeneous mechanistic scope of the review. Comparisons among mechanistic patterns were descriptive and based on supporting-study volume, observed consistency, validation characteristics, and component-specific limitations. They should not be interpreted as GRADE ratings or as validated categories of evidentiary certainty. **Table 3** therefore summarizes the relative pattern of support and its limitations without assigning an overall confidence grade.

**Table 3:** Narrative comparison of support for hypoxia-driven CSC–immune reprogramming patterns.

Mechanistic route	Observed consistency	Validation characteristics	Supporting evidence volume	Descriptive synthesis and limitations
Cytotoxic effector-cell escape	Recurrent across several studies	Functional NK-, CIK-, CD8^+^ T-cell, and CTL assays; some animal validation	Moderate	Convergent findings linked hypoxic CSC-like states with impaired immune recognition or killing. Interpretation is limited by marker-based CSC assessment in some models and restricted human validation.
Myeloid/macrophage-mediated CSC support	Recurrent across several studies	Cell, macrophage, animal, tissue, EV, vascular, and dataset-supported evidence	Moderate	Multiple models linked hypoxia-associated myeloid remodeling with CSC plasticity or support. The evidence remains predominantly preclinical, and direct functional immune validation was incomplete in some cancer contexts.
Checkpoint-associated and adaptive immune suppression	Present but more heterogeneous	Checkpoint expression, Treg/MDSC findings, selected T-cell assays, and immunotherapy-response models	Small to moderate	Evidence involved PD-L1, CD47, Tregs, MDSCs, and CD8^+^ T-cell suppression. Some findings were based on surrogate markers or therapeutic reversal rather than endogenous immune-function experiments.
Cross-cutting hypoxic niche phenotype	Broad qualitative convergence	Variable combinations of hypoxia, stemness, immune, vascular, and metabolic measurements	Moderate	Related features recurred across tumors, but cancer types, hypoxia models, CSC definitions, immune endpoints, and validation depth were heterogeneous; the niche is therefore a mechanistic framework rather than a formally graded universal pathway.
Interventional/translational targeting of hypoxic CSC–immune niches	Supported by a small number of intervention studies	Hypoxia-responsive or hypoxia-relieving platforms with CSC, MDSC/PD-L1, T-cell, anti-PD-1, or immune-memory outcomes	Small	The studies indicate potential modifiability of selected compartments, but therapeutic perturbation is not equivalent to evidence of spontaneous endogenous niche formation or established clinical efficacy.

Future studies should move from isolated pathway analysis toward spatial and functional validation of the complete axis. Priority approaches include multiplex immunohistochemistry, spatial transcriptomics, single-cell multi-omics, hypoxia mapping, patient-derived organoids, autologous tumor-immune co-culture systems, and treatment-response-linked cohorts. These approaches would help determine whether hypoxic CSC–immune niches are detectable in patient tumors, whether they predict resistance to immunotherapy or other treatments, and whether they can be safely disrupted. Future work should also clarify whether specific mechanisms are cancer-type restricted or represent broader features of hypoxic, stemness-permissive, immune-refractory tumor niches.

Future studies should also separate three levels of evidence that are often merged in the literature: coexistence, spatial association, and functional interaction. Coexistence means that hypoxia, CSC markers, and immune-suppressive features are present in the same tumor. Spatial association means that these features localize to the same tumor regions. Functional interaction means that perturbing one component, such as hypoxia-associated signaling, changes CSC behavior and immune function in a linked manner. The strongest future evidence would demonstrate all three levels, ideally using patient-derived models and spatially resolved human tissue validation (8, 14, 36).

A practical next step would be to design studies that begin with spatial mapping of human tumors, identify hypoxic regions enriched for CSC and immune-suppressive markers, and then test matched patient-derived models for immune resistance and therapeutic vulnerability. Such studies could compare hypoxic and non-hypoxic tumor regions from the same patient, examine whether CSC-like populations show differential sensitivity to NK-cell or CD8-positive T-cell killing, and determine whether macrophage, MDSC, or checkpoint-associated signals mediate this resistance. Linking these findings to clinical response would be particularly valuable (14, 15). If hypoxic CSC–immune niches predict poor response to immune checkpoint blockade, chemotherapy, radiotherapy, or combined approaches, they could become useful biomarkers for treatment stratification and rational combination therapy design.

This would move the field beyond descriptive association and toward clinically relevant validation of the niche framework. For this reason, future work should prioritize integrated experimental designs rather than isolated assessment of hypoxia, CSC markers, or immune endpoints. The most informative studies will be those that test whether hypoxic CSC–immune niches are spatially identifiable, functionally immune-resistant, therapeutically modifiable, and clinically predictive.

## 5 Conclusion

This systematic review supports hypoxia as a coordinating microenvironmental pressure linking CSC plasticity with immune dysfunction in selected tumor contexts. Across 14 included studies, the evidence converged around cytotoxic effector-cell escape, myeloid/macrophage-mediated CSC support, checkpoint-associated and adaptive immune suppression, and a broader cross-cutting hypoxic niche phenotype. Together, these patterns suggest that localized hypoxic tumor regions may become stemness-permissive, immune-refractory, and therapy-resistant. Cytotoxic immune dysfunction and myeloid/macrophage-mediated CSC support recurred across several experimental platforms, whereas checkpoint-associated and adaptive immune suppression was more heterogeneous and was sometimes supported by surrogate or interventional evidence. Interventional studies further suggest that selected hypoxic CSC–immune compartments may be therapeutically modifiable through approaches that reduce CSC-like populations, attenuate MDSC- or PD-L1-associated immune escape, and improve antitumor immune responses. However, the proposed hypoxic CSC–immune niche remains a context-dependent mechanistic framework and should not be interpreted as evidence of a universal pathway across cancers. Spatially resolved human tumor studies, patient-derived tumor–immune systems, single-cell and spatial profiling, and treatment-response-linked cohorts are needed to establish clinical relevance, predictive value, and safe therapeutic targeting.

## Data Availability

The original contributions presented in the study are included in the article/**Supplementary Material**. Further inquiries can be directed to the corresponding author/s.

## Glossary

AKT: Protein kinase B
ALDH: Aldehyde dehydrogenase
ALDH1: Aldehyde dehydrogenase 1
ATRA: All-trans retinoic acid
BCSC: Breast cancer stem cell
BMDC: Bone marrow-derived dendritic cell
BRCA1-IRIS: BRCA1 intron 11 retaining isoform
CAIX: Carbonic anhydrase IX
CD: Cluster of differentiation
CIK: Cytokine-induced killer
CoCl₂: Cobalt chloride
CSC: Cancer stem cell
CRT: Calreticulin
CTL: Cytotoxic T lymphocyte
CuET: Bis(diethyldithiocarbamate)-copper
DDA: Docosadienoic acid
EMT: Epithelial-mesenchymal transition
EV: Extracellular vesicle
FACS: Fluorescence-activated cell sorting
FADS1: Fatty acid desaturase 1
Fzd7: Frizzled-7
GM-CSF: Granulocyte-macrophage colony-stimulating factor
GSC: Glioma stem cell
HIF: Hypoxia-inducible factor
HIF-1α: Hypoxia-inducible factor-1 alpha
HIF-2α: Hypoxia-inducible factor-2 alpha
HMGB1: High mobility group box 1
ICAM-1: Intercellular adhesion molecule 1
ICD: Immunogenic cell death
ICG: Indocyanine green
IF: Immunofluorescence
IFN-γ: Interferon gamma
IHC: Immunohistochemistry
IL-10: Interleukin-10
MDSC: Myeloid-derived suppressor cell
MICA: MHC class I chain-related protein A
mTOR: Mechanistic target of rapamycin
NANOG: NANOG homeobox
NF-κB: Nuclear factor kappa B
NK: Natural killer
NKG2D: Natural killer group 2 member D
NOS: Newcastle-Ottawa Scale
NSCLC: Non-small cell lung cancer
OCT4: Octamer-binding transcription factor 4
OSA: Obstructive sleep apnea
PD-1: Programmed death 1
PD-L1: Programmed death-ligand 1
PDT: Photodynamic therapy
PGCC: Polyploid giant cancer cell
PRISMA: Preferred Reporting Items for Systematic Reviews and Meta-Analyses
PROSPERO: International Prospective Register of Systematic Reviews
SMAD: SMAD family transcription factor
SNHG16: Small nucleolar RNA host gene 16
SOX2: SRY-box transcription factor 2
SLC16A1: Solute carrier family 16 member 1
SYRCLE: Systematic Review Centre for Laboratory Animal Experimentation
TAM: Tumor-associated macrophage
TGFBI: Transforming growth factor beta induced
TGF-β: Transforming growth factor beta
TGF-β1: Transforming growth factor beta 1
TGF-β2: Transforming growth factor beta 2
TLR2: Toll-like receptor 2
TME: Tumor microenvironment
TNBC: Triple-negative breast cancer
Tregs: Regulatory T cells
TREM1: Triggering receptor expressed on myeloid cells 1
VEGFA: Vascular endothelial growth factor A
Wnt/β-catenin: Wingless/Int-1 beta-catenin
