# Supplementary material for: Hypoxic cancer stem cell–immune niches in the tumor microenvironment: a Systematic Review of mechanisms and therapeutic implications
Source: Front Immunol. 2026 Sep 22;17:1942424. doi: 10.3389/fimmu.2026.1942424 (PMC13638562; doi:10.3389/fimmu.2026.1942424)
Supplement: Supplementary file 1 [file Table1.docx]

**Supplementary Data Sheet 1. Database-specific search strategies**

This supplementary data sheet reports the database-specific search strategies used for the systematic review, including search dates, date ranges, language limits, full search strings, and records retrieved.

**Search Summary**

| **Database/source** | **Platform** | **Date searched** | **Date range** | **Language limit** | **Records retrieved** |
| --- | --- | --- | --- | --- | --- |
| PubMed | PubMed/MEDLINE | 7 February 2026 | 1 January 2010 to 31 January 2026 | English | 203 |
| Embase | Embase | 7 February 2026 | 1 January 2010 to 31 January 2026 | English | 499 |
| Scopus | Scopus | 8 February 2026 | 1 January 2010 to 31 January 2026 | English | 104 |
| Web of Science | Web of Science Core Collection | 8 February 2026 | 1 January 2010 to 31 January 2026 | English | 163 |
| Google Scholar | Google Scholar | 9 February 2026 | 1 January 2010 to 31 January 2026 | English | 600 recorded before deduplication; the first 600 relevance-ranked records were screened. Two parallel reviewer teams each collectively covered the complete 600-record set, with the workload divided within each team; each record therefore received one assessment from each team. |

**PubMed**

| **Database** | **PubMed/MEDLINE** |
| --- | --- |
| Date searched | 7 February 2026 |
| Search period | 1 January 2010 to 31 January 2026 |
| Language | English |
| Records retrieved | 203 |

**Full Search String**

("Hypoxia"[MeSH Terms] OR "hypoxi*"[Title/Abstract] OR "HIF-1alpha"[Title/Abstract] OR "HIF-1α"[Title/Abstract] OR "HIF-2alpha"[Title/Abstract] OR "HIF-2α"[Title/Abstract] OR "CAIX"[Title/Abstract]) AND ("Neoplastic Stem Cells"[MeSH Terms] OR "cancer stem cell*"[Title/Abstract] OR "tumor stem cell*"[Title/Abstract] OR "tumor-initiating cell*"[Title/Abstract] OR "sphere formation"[Title/Abstract] OR "CSC"[Title/Abstract]) AND ("Tumor Escape"[MeSH Terms] OR "immune evasion"[Title/Abstract] OR "immune escape"[Title/Abstract] OR "immunosuppress*"[Title/Abstract] OR "tumor microenvironment"[Title/Abstract] OR "immunotherapy resistance"[Title/Abstract] OR "immune checkpoin*"[Title/Abstract]) NOT ("Review"[Publication Type] OR "Systematic Review"[Publication Type] OR "Meta-Analysis"[Publication Type] OR "Scoping Review"[Publication Type] OR "Case Reports"[Publication Type] OR "Editorial"[Publication Type] OR "Letter"[Publication Type] OR "Comment"[Publication Type] OR "News"[Publication Type] OR "Clinical Trial Protocol"[Publication Type] OR "systematic review"[Title/Abstract] OR "meta-analysis"[Title/Abstract])

Filters applied: English language; publication date from 1 January 2010 to 31 January 2026.

Sort: Publication date.

**Embase**

| **Database** | **Embase** |
| --- | --- |
| Date searched | 7 February 2026 |
| Search period | 1 January 2010 to 31 January 2026 |
| Language | English |
| Records retrieved | 499 |

**Full Search String**

(

('hypoxia'/exp OR hypoxi*:ti,ab,kw OR 'hif-1alpha':ti,ab,kw OR 'hif-1a':ti,ab,kw OR 'hif-2alpha':ti,ab,kw OR 'hif-2a':ti,ab,kw OR 'caix':ti,ab,kw)

AND

('cancer stem cell'/exp OR 'cancer stem cell*':ti,ab,kw OR 'tumor stem cell*':ti,ab,kw OR 'tumor-initiating cell*':ti,ab,kw OR 'sphere formation':ti,ab,kw OR 'csc':ti,ab,kw)

AND

('immune evasion'/exp OR 'tumor microenvironment'/exp OR 'immune evasion':ti,ab,kw OR 'immune escape':ti,ab,kw OR immunosuppress*:ti,ab,kw OR 'tumor microenvironment':ti,ab,kw OR 'immunotherapy resistance':ti,ab,kw OR 'immune checkpoint*':ti,ab,kw)

)

NOT

('review'/it OR 'systematic review'/it OR 'meta analysis'/it OR 'case report'/it OR 'editorial'/it OR 'letter'/it OR 'conference abstract'/it OR 'systematic review':ti,ab,kw OR 'meta-analysis':ti,ab,kw)

AND [english]/lim AND [2010-2026]/py

Limits applied: English language; publication date 1 January 2010 to 31 January 2026.

**Scopus**

| **Database** | **Scopus** |
| --- | --- |
| Date searched | 8 February 2026 |
| Search period | 1 January 2010 to 31 January 2026 |
| Language | English |
| Records retrieved | 104 |

**Full Search String**

TITLE-ABS-KEY ( ( hypoxi* OR "hypoxia inducible factor" OR "HIF-1α" OR "HIF-1alpha" OR "HIF-2α" OR "HIF-2alpha" ) AND ( "cancer stem cell*" OR "tumor stem cell*" OR "tumor-initiating cell*" ) AND ( "immune evasion" OR "immune escape" OR immunosuppress* OR "immune checkpoint*" OR "immunotherapy resistance" ) ) AND PUBYEAR > 2009 AND PUBYEAR < 2027 AND ( LIMIT-TO ( DOCTYPE , "ar" ) ) AND ( LIMIT-TO ( LANGUAGE , "English" ) )

**Web of Science**

| **Database** | **Web of Science Core Collection** |
| --- | --- |
| Date searched | 8 February 2026 |
| Search period | 1 January 2010 to 31 January 2026 |
| Language | English |
| Records retrieved | 163 |

**Full Search String**

TS = (

(

hypoxi*

OR "hypoxia inducible factor"

OR "HIF-1alpha"

OR "HIF-1α"

OR "HIF-2alpha"

OR "HIF-2α"

OR CAIX

)

AND

(

"cancer stem cell*"

OR "tumor stem cell*"

OR "tumor-initiating cell*"

OR "sphere formation"

OR CSC

)

AND

(

"immune evasion"

OR "immune escape"

OR immunosuppress*

OR "tumor microenvironment"

OR "immunotherapy resistance"

OR "immune checkpoint*"

)

)

Limits applied: English language; publication date 1 January 2010 to 31 January 2026; document type Article

**Google Scholar**

| **Source** | **Google Scholar** |
| --- | --- |
| Date searched | 9 February 2026 |
| Search period | 1 January 2010 to 31 January 2026 |
| Language | English |
| Records retrieved/recorded | 600 recorded before deduplication; the first 600 relevance-ranked records were screened. Two parallel reviewer teams each collectively covered the complete 600-record set, with the workload divided within each team; each record therefore received one assessment from each team. |

**Full Search String**

Google Scholar does not reliably support complex reproducible Boolean strings across sessions. Searches were therefore documented as concept-based phrase searches and screened by relevance ranking. The first 600 relevance-ranked records were recorded before duplicate removal and screened within the same two-team process used for the complete record set. Each reviewer team collectively covered all 600 records, with the workload divided between its two members, so every record received one assessment from each team.

Search phrases used:

"hypoxia" "cancer stem cells" "immune escape" "tumor microenvironment"

"hypoxia" "stemness" "immune suppression" cancer

"HIF-1α" "cancer stem cells" "PD-L1"

"HIF-1α" "cancer stem cells" macrophage

"hypoxia" "cancer stem cells" "natural killer cells"

"hypoxia" "stemness" Tregs cancer

"hypoxia" "cancer stem cells" "tumor-associated macrophages"

"hypoxia" "cancer stem cells" immunosuppression

Limits applied: English language; publication date 1 January 2010 to 31 January 2026 where Google Scholar filtering allowed.

Supplementary identification: Reference lists of relevant articles were examined to identify potentially eligible reports. Reports identified in this way were assessed against the same eligibility criteria as database-derived records.

**Filters and limits**

Filters applied across database searches were English language and publication date from 1 January 2010 to 31 January 2026. Publication-type or document-type limits were applied where specified in the database-specific search strings. Searches were restricted to English-language records published from 1 January 2010 to 31 January 2026.

**Duplicate Management**

Retrieved records were exported to Zotero, where duplicates were identified and manually confirmed before removal. Deduplicated records were then imported into Rayyan for title and abstract screening.

**Record counts used for PRISMA reporting**

The search yielded 1,569 records before duplicate removal: PubMed (n = 203), Web of Science (n = 163), Embase (n = 499), Scopus (n = 104), and Google Scholar (n = 600). After removal of 416 duplicates, 1,153 records proceeded to title and abstract screening.
