# Supplementary material for: Hypoxic cancer stem cell–immune niches in the tumor microenvironment: a Systematic Review of mechanisms and therapeutic implications
Source: Front Immunol. 2026 Sep 22;17:1942424. doi: 10.3389/fimmu.2026.1942424 (PMC13638562; doi:10.3389/fimmu.2026.1942424)
Supplement: Supplementary file 2 [file Table2.docx]

**Supplementary Data Sheet 2. Detailed study characteristics and extracted evidence of included studies**

| **Study** | **Country** | **Cancer type** | **Study model** | **Hypoxia exposure/mechanism** | **CSC/stemness evidence** | **Immune component/outcome** | **Proposed CSC-immune mechanism** | **Key finding** | **Translational relevance** |
| --- | --- | --- | --- | --- | --- | --- | --- | --- | --- |
| Wang et al., 2025 | China | Triple-negative breast cancer | Mixed: in vitro TNBC models, NK-cell assays, xenograft model, human tissue | 1% O₂; HIF-1α activation; Akt/Wnt/β-catenin signaling | CD44+/CD24-, ALDH1+, Frizzled-7 expression, CSC enrichment | NK cells, NKG2D, MICA, CD107a, granzyme B, perforin, CCL5, CXCL9 | Hypoxia activates HIF-1α/Akt/Wnt/β-catenin signaling, enriching Fzd7-positive CSC-like cells and impairing MICA–NKG2D-mediated NK recognition | Hypoxic Fzd7-positive CSC-like TNBC cells showed reduced MICA–NKG2D-mediated NK-cell recognition, while the Fzd7-targeting SHH002-hu1-MICA fusion protein enhanced NK-cell retargeting and cytotoxic activation in experimental models. | Supports Fzd7/MICA–NKG2D-directed strategies, including SHH002-hu1-MICA-like fusion approaches, to restore NK-cell surveillance against hypoxic CSC-like TNBC populations. |
| Geng et al., 2024 | China | Colorectal cancer | In vitro CSC and NK-cell co-culture | Hypoxic exposure; FADS1/DDA-axis activation; increased spermine levels | CD44, CD133, colorectal CSC phenotype | NK cells, IFN-γ, TNF-α, IL-6, NKG2D, NKp30, NKp44, NKp46, KIR2DL1, KIR3DL1, NK cytotoxicity, NK apoptosis, spermine | Hypoxia-associated metabolic remodeling in colorectal CSCs involved FADS1/DDA-axis activation and increased spermine levels, which were associated with reduced NK-cell cytokine secretion, impaired cytotoxicity, and increased NK-cell apoptosis. | Hypoxic colorectal CSCs evaded NK-mediated immune attack through metabolic changes involving FADS1/DDA signaling and spermine-associated suppression of NK-cell function. | Suggests metabolic targeting of FADS1/DDA signaling and spermine-associated NK-cell suppression to restore antitumor NK function. |
| Dong et al., 2024 | China | Glioblastoma | Mixed: patient samples, public datasets, cell lines, macrophage assays, orthotopic xenograft | 1% O₂; HIF-1α-mediated transcriptional regulation of TREM1 | CD133, SOX2, CD15, Olig2, CD44, tumorsphere formation, mesenchymal-like transition | Tumor-associated macrophages, M2-like TAMs, CD163, CD206, TREM1, TLR2, TGF-β2, IL-8, IL-10, MMP9, VEGF | Hypoxia-induced HIF-1α upregulates TREM1 in TAMs, activating TLR2/AKT/mTOR/c-MYC signaling and TGF-β2 secretion, which promotes mesenchymal-like glioma stem cell transition. | Hypoxia-conditioned TAM signaling supported GSC plasticity and mesenchymal-like transition. | Suggests targeting the TREM1–TLR2/AKT/mTOR/c-MYC–TGF-β2 axis to disrupt CSC-supportive glioblastoma niches. |
| Sami et al., 2020 | USA | Triple-negative breast cancer | Mixed: TNBC cell models, animal models, human samples | 1% O₂; BRCA1-IRIS overexpression; HIF-1α/NF-κB activation; GM-CSF induction | CD44+/CD24-, SOX2, OCT4, NANOG, EMT markers including CDH2, Twist, and SnailI | TAMs, M1/M2 polarization, GM-CSF, TGF-β1, PD-L1, CD47, calreticulin, CD8+/PD-1+ T cells, CD25+/FOXP3+ Tregs | In BRCA1-IRIS-overexpressing TNBC, hypoxia-associated HIF-1α/NF-κB signaling promotes GM-CSF secretion, TAM recruitment/polarization, and TGF-β1-mediated CSC/EMT traits, alongside Treg infiltration, CD8+/PD-1+ T-cell exhaustion, PD-L1 upregulation, and CD47-mediated immune escape. | BRCA1-IRIS-overexpressing hypoxic TNBC cells established a GM-CSF/TAM/TGF-β1 feedback loop that promoted CSC/EMT traits, checkpoint-associated immune escape, Treg enrichment, and CD8+ T-cell exhaustion. | Supports IRIS-targeted disruption of tumor–TAM crosstalk, including IRISpep-like approaches, to reduce CSC/EMT traits, PD-L1/CD47-mediated immune escape, and restore antitumor immune surveillance. |
| Hasmim et al., 2013 | France | Melanoma | Mixed: melanoma cell model and mouse tumor model | 1% O₂; HIF-1α-dependent NANOG induction | NANOG, ALDH activity, spheroid formation, tumorigenic potential | CD4+CD25+FOXP3+ Tregs, F4/80+ macrophages, CD3+CD8+ T cells, TGF-β1, IL-10, arginase-1 | Hypoxia induces HIF-1α-dependent NANOG, which regulates TGF-β1 and promotes Treg and macrophage infiltration, arginase-1-associated immunosuppression, and reduced CD8+ T-cell activity. | Hypoxia-induced NANOG linked stemness features with TGF-β1-mediated Treg/macrophage recruitment and CD8+ T-cell suppression; NANOG targeting enhanced the antitumor effect of TRP-2 peptide vaccination in the melanoma model. | Supports targeting the NANOG/TGF-β1 axis to improve immune control and potentially enhance peptide vaccine-based antitumor responses in hypoxic melanoma models. |
| Bu et al., 2019 | China | Epithelial ovarian cancer | Ex vivo/in vitro: ovarian cancer spheroids and human-derived CIK cells. | CoCl₂-mediated HIF-1α stabilization; 3D spheroid culture | ALDH1A1, NANOG, spheroid formation, anoikis resistance. | Human-derived CIK cells, CD3+CD56+ cytotoxic effector cells, ICAM-1, TGF-β1/SMAD, NF-κB, VEGFA. | HIF-1α-associated TGF-β1/SMAD and VEGFA signaling downregulates ICAM-1, reducing CIK-cell binding and lysis of ovarian CSC-like spheroids. | Ovarian CSC-like spheroids showed resistance to CIK-cell-mediated cytotoxicity under hypoxia-associated conditions. | Suggests that targeting the HIF-1α/TGF-β1/ICAM-1 axis may improve CIK-cell-mediated cytotoxicity against ovarian CSC-like spheroids. |
| Liu et al., 2022 | China | Glioblastoma | Mixed: glioma cell model, macrophage assays, public datasets, small human tissue validation | CoCl₂-mediated chemical hypoxia; HIF-1α stabilization | CD133, Nestin, OCT4, polyploid giant cancer cells, stem-like progeny, tumorspheres | TAMs, M1/M2 markers, IL-10, TNF-α, CD163, CD80 | CoCl₂-induced HIF-1α stabilization promotes PGCC formation and stem-like progeny, which support M2-like TAM polarization. | Hypoxia-induced PGCC-derived progeny supported tumor-promoting macrophage transformation | Suggests targeting hypoxia-induced PGCCs or their stem-like progeny as a potential strategy to limit glioma progression and macrophage-mediated immune suppression. |
| Yin et al., 2022 | China | Endometrial cancer | Mixed: endometrial cancer stem-like cells and xenograft model | 2% O₂; CoCl₂; HIF-1α/HIF-2α-mediated PD-L1 regulation | CD133, ALDH1, OCT4, SOX2, NANOG, sphere formation, colony formation, tumorigenicity | PD-L1; PD-1/PD-L1 axis discussed as checkpoint-relevant context | HIF-1α/HIF-2α activate PD-L1 transcription in stem-like endometrial cancer cells, supporting PD-L1-associated CSC self-renewal and tumorigenicity; checkpoint-mediated immune escape was biologically implicated but not directly tested with immune effector cells. | Hypoxia-induced HIF-1α/HIF-2α upregulated PD-L1, which supported CSC self-renewal, pluripotency marker expression, colony/sphere formation, and tumorigenicity. | Suggests checkpoint-informed targeting of hypoxic CSC-like populations; however, immune-effector validation was absent/limited because functional T-cell or immune co-culture assays were not performed. |
| Hou et al., 2025 | China | Non-small cell lung cancer | Mixed: NSCLC patient tissues and serum EVs, A549 and H1299 NSCLC cell models, THP-1 macrophage polarization assays, EV co-culture system, and A549 tumor-bearing mouse model | Hypoxic stimulation of NSCLC cells; HIF-1α activation; hypoxic NSCLC-derived extracellular vesicles enriched with SNHG16 | SOX2, OCT4, NANOG, CSC-like properties, EMT-associated changes, tumor growth | M2 macrophage polarization, THP-1 cells, CD163, CD206, Arg-1, IL-10, TGF-β, VEGF, IL-1β, iNOS | Hypoxic NSCLC cells release SNHG16-containing extracellular vesicles that promote CSC-like properties and M2 macrophage polarization through the SNHG16/miR-132-3p/KIF5A axis. | Hypoxic NSCLC-derived SNHG16-enriched EVs enhanced NSCLC proliferation, EMT, CSC-related gene expression, tumor growth, and M2 macrophage polarization; these effects were reduced by SNHG16 silencing, miR-132-3p overexpression, or KIF5A inhibition. | Suggests that targeting hypoxic EV-mediated SNHG16/miR-132-3p/KIF5A signaling may disrupt CSC-like aggressiveness and macrophage-mediated immune remodeling in NSCLC. |
| Liu et al., 2025 | China | Breast cancer / 4T1 tumor model | Mixed: 4T1 cell model, BCSC-related assays, 3D tumor spheroids, ApoBD-mediated deep tumor penetration model, 4T1 and 4T1/DOX tumor-bearing mouse models, and anti-PD-1 combination therapy | Hypoxia-responsive nitroimidazole-modified nanocore; HIF-1α-positive hypoxic tumor regions; hypoxia-associated enrichment of CSCs and MDSCs; ATRA release in hypoxic deep tumor sites | CD133, CD44+/CD24− BCSC population, SP cells, tumorsphere formation, SOX2, OCT4, NANOG | MDSCs, Gr-1, CD8+ T cells, anti-PD-1 response, immunosuppressive microenvironment, chemoimmunotherapy response | In an interventional model, hypoxia-responsive HRA@D-TT delivered ATRA into deep hypoxic tumor regions to promote differentiation of CSCs and MDSCs, thereby reducing chemoresistance and immunosuppression and improving anti-PD-1-based chemoimmunotherapy. | HIF-1α-positive hypoxic tumor regions were associated with CD133-positive CSCs and Gr-1-positive MDSCs, while HRA@D-TT reduced CSC and MDSC compartments and enhanced chemotherapy and immune checkpoint blockade efficacy. | Supports hypoxia-targeted differentiation therapy as a strategy to jointly target CSCs and MDSCs in poorly accessible hypoxic tumor regions and improve chemoimmunotherapy. |
| Zhang et al., 2025 | China | Triple-negative breast cancer / 4T1 tumor model | Mixed: hypoxic 4T1 cell assays, CD24+CD44+ALDH1A1+ CSC assessment, T-cell killing assay, BMDC maturation assay, 4T1 tumor-bearing mouse model, immune profiling, transcriptomic analysis, and tumor rechallenge model | Hypoxic 4T1 culture; reduced oxygen consumption by CuET/ICG nanoparticles; alleviation of cellular and tumor hypoxia; AMPK activation under hypoxia; HIF-1α/hypoxia assessment in tumor model | CD24+CD44+ALDH1A1+ CSCs, CSC-associated PD-L1, stem cell population maintenance pathway, reduced CSCs after combination therapy | PD-L1, T-cell killing, CRT, HMGB1, BMDC maturation, CD8+ T-cell infiltration, IFNγ+ CD8+ T cells, Tregs, immune memory | In an interventional model, CuET/ICG nanoparticles reduced oxygen consumption and relieved hypoxia, activated AMPK-mediated degradation of membrane PD-L1 on tumor cells and CSCs, and improved T-cell-mediated antitumor immunity. | CuET/ICG nanoparticles combined with PDT relieved hypoxia, amplified immunogenic cell death, reduced membrane PD-L1 on 4T1 cells and CSCs, improved T-cell killing, increased DC maturation and CD8+ T-cell responses, reduced Tregs, and promoted immune memory. | Suggests that hypoxia-relieving mitochondrial interference may overcome PD-L1-mediated immune evasion in CSC-containing TNBC and enhance antitumor immunotherapy. |
| Jang et al., 2022 | South Korea / USA | Pancreatic cancer | Mixed: in vitro TME mimic system using pancreatic cancer cells, fibroblasts/CAFs, U937-derived macrophages, hypoxia and gemcitabine stress, derivative cell isolation, multi-omic analysis, functional assays, and TCGA/GEO survival analysis | Hypoxic and gemcitabine-stressed TME mimic; pancreatic cancer TME characterized by fibrosis and hypoxia; hypoxia-associated stress condition combined with chemotherapy | CD44high/SLC16A1high derivative cells, ALDH activity, tumorsphere formation, anchorage-independent growth, invasiveness, CD24-associated phenotype transition | Macrophages, U937-derived macrophages, macrophage-to-cancer-cell direct transfer of cellular components, TME cell-to-cell transfer | Under hypoxic and chemotherapy-stressed TME conditions, macrophage-to-pancreatic cancer cell transfer generated derivative cells with CSC-like, invasive, and metabolically reprogrammed phenotypes. | Stressed TME conditions increased direct cell-to-cell transfer from macrophages to pancreatic cancer cells, producing CD44high/SLC16A1high derivative cells with enhanced tumorsphere formation, anchorage-independent growth, invasiveness, and adverse prognostic association. | Suggests that blocking macrophage-associated direct cell-to-cell transfer, CD44, or MCT1/SLC16A1 may help limit chemotherapy-induced tumor evolution and recurrence in hypoxic pancreatic cancer. |
| Fico and Santamaria-Martínez, 2020 | Switzerland | Breast cancer | Mixed: MMTV-PyMT;Tgfbi knockout mouse model, tumor sphere assays, FACS analysis, limiting dilution/tumor-initiation assays, lung metastasis assessment, vascular perfusion and leakiness assays, pimonidazole hypoxia staining, bone marrow transplantation, human breast cancer tissue analysis, and METABRIC/CIBERSORT analysis | TGFBI-dependent tumor vascular dysfunction; reduced angiogenesis and hypoxia signatures after TGFBI deletion; improved vessel perfusion; reduced vascular leakiness; decreased pimonidazole-positive hypoxic areas | Mammosphere/tumor sphere formation, ALDHhigh cells, Aldh1a3, CD24+CD90+ metastatic CSCs, tumor-initiating capacity | CD11b+ cells, TAMs, myeloid cells, M2 macrophages, CD8+ T cells, immune compartment analysis, CIBERSORT | TGFBI from macrophage/stromal compartments promotes abnormal tumor vasculature and hypoxia, creating a CSC-permissive and metastasis-supportive microenvironment associated with myeloid/M2 enrichment and reduced CD8+ T-cell content. | TGFBI deletion normalized tumor vasculature, reduced tumor hypoxia, decreased CSC content and lung metastasis, while high TGFBI expression in human breast cancer correlated with poor prognosis, aggressive subtypes, myeloid/M2 macrophage enrichment, and lower CD8+ T-cell content. | Supports TGFBI and stromal/myeloid vascular-hypoxia remodeling as potential targets to reduce CSC-supported metastasis and improve therapeutic delivery in breast cancer. |
| Akbarpour et al., 2017 | USA / Spain | Epithelial lung tumor / TC1 tumor model | In vivo TC1 murine epithelial lung tumor model exposed to intermittent hypoxia or sleep fragmentation, with flow cytometry, qPCR, histological invasion assessment, and ex vivo CTL cytolytic activity assay | Intermittent hypoxia exposure mimicking obstructive sleep apnea; repeated 6% to 21% FiO₂ cycles; sleep fragmentation as parallel OSA-related stressor | Oct4+ CSC-like cells, CD44+CD133+ CSC-like cells, CSC marker-positive tumor populations | CD8+ T cells, tumor-infiltrating lymphocytes, granzyme B, perforin, IFN-γ, Tregs, CTL-mediated tumor-cell killing | Intermittent hypoxia and sleep fragmentation altered the tumor microenvironment by impairing CD8+ cytotoxic T-cell function while increasing CSC marker-positive tumor populations, supporting reduced immunosurveillance and enhanced tumor aggressiveness. | Intermittent hypoxia and sleep fragmentation increased tumor growth and invasion; intermittent hypoxia reduced granzyme B-producing intratumoral CD8+ T cells, both exposures reduced GzmB, perforin, IFN-γ and CTL killing, and CSC marker-positive tumor populations increased. | Provides mechanistic support that OSA-related intermittent hypoxia may worsen tumor behavior by combining impaired cytotoxic immunosurveillance with expansion of CSC-like tumor populations. |
