# Supplementary material for: Hypoxic cancer stem cell–immune niches in the tumor microenvironment: a Systematic Review of mechanisms and therapeutic implications
Source: Front Immunol. 2026 Sep 22;17:1942424. doi: 10.3389/fimmu.2026.1942424 (PMC13638562; doi:10.3389/fimmu.2026.1942424)
Supplement: Supplementary file 3 [file Table3.docx]

**Supplementary Data Sheet 3. Component-specific methodological appraisal of included studies**

Appraisal was conducted separately for each applicable evidence component. Domain-level judgments were used to identify methodological strengths and limitations; no numerical scoring, cross-domain aggregation, or combined overall study-level rating was performed.

**Supplementary Table S1. SYRCLE risk of bias assessment for animal experimental components**

| **Study** | **Animal component assessed** | **Random allocation** | **Allocation concealment** | **Blinding of investigators or outcome assessment** | **Incomplete outcome data** | **Selective outcome reporting** | **Other sources of bias** | **Justification** |
| --- | --- | --- | --- | --- | --- | --- | --- | --- |
| Wang et al., 2025 | TNBC xenograft model evaluating SHH002 hu1 MICA and NK mediated antitumor activity | Low risk | Unclear risk | Unclear risk | Low risk | Unclear risk | Moderate concern | Mice were randomized into treatment groups, with n equals 5 per group, but the method of sequence generation, allocation concealment, and blinding were not clearly reported. Attrition was not evident from the reported methods. |
| Dong et al., 2024 | Orthotopic glioma stem cell xenograft model with U937 derived macrophage co implantation | Unclear risk | Unclear risk | Unclear risk | Low risk | Unclear risk | Moderate concern | Animal experiments were approved and an orthotopic model was used, but randomization, allocation concealment, and blinding procedures were not clearly described. The model was biologically relevant but depended on co implanted macrophage systems. |
| Sami et al., 2020 | Orthotopic and syngeneic mammary tumor models evaluating IRIS driven TAM interaction and immune suppression | Unclear risk | Unclear risk | Unclear risk | Low risk | Unclear risk | Moderate concern | Animal experiments were approved and multiple in vivo models were used, but randomization and blinding were incompletely reported. The multi model design strengthens biological validity, but internal validity remains limited by incomplete reporting of bias reduction procedures. |
| Hasmim et al., 2013 | B16 F10 melanoma mouse model with Nanog targeting and vaccination experiments | Unclear risk | Unclear risk | Unclear risk | Low risk | Unclear risk | Moderate concern | The study used an in vivo melanoma model with immune infiltration assessment, but randomization, allocation concealment, and blinding were not clearly reported. Mechanistic validation was strong, but reporting of animal bias safeguards was limited. |
| Yin et al., 2022 | Endometrial cancer stem like cell xenograft model evaluating PD-L1 knockdown and tumorigenicity | Low risk | Unclear risk | Low to unclear risk | Low risk | Unclear risk | Moderate concern | The paper reported random assignment and blinded grouping of mice, which lowers selection and performance concerns. However, allocation concealment and outcome assessor blinding were not fully detailed, and the animal work relied mainly on a limited endometrial cancer stem like cell model. |
| Hou et al., 2025 | A549 tumor-bearing mouse model evaluating hypoxic NSCLC-derived SNHG16-containing EVs, tumor growth, CSC-related markers, and M2 macrophage polarization | Unclear risk | Unclear risk | Unclear risk | Low risk | Unclear risk | Moderate concern | The study included an in vivo tumor model supporting EV-mediated NSCLC aggressiveness and macrophage polarization, but randomization, allocation concealment, blinding, and sample-size justification were not clearly reported. Attrition was not evident from the available methods. |
| Liu et al., 2025 | 4T1 and 4T1/DOX tumor-bearing mouse models evaluating hypoxia-responsive HRA@D-TT, CSC/MDSC differentiation, chemotherapy, and anti-PD-1 response | Unclear risk | Unclear risk | Unclear risk | Low risk | Unclear risk | Moderate concern | Multiple in vivo treatment models were used, including chemoimmunotherapy evaluation, but the study was primarily a therapeutic nanoplatform design. Randomization, allocation concealment, and blinding procedures were incompletely described. |
| Zhang et al., 2025 | 4T1 tumor-bearing mouse model evaluating CuET/ICG nanoparticles, PDT, tumor hypoxia relief, PD-L1 downregulation, antitumor immunity, and immune memory | Unclear risk | Unclear risk | Unclear risk | Low risk | Unclear risk | Moderate concern | The animal work included tumor immune profiling and rechallenge experiments, strengthening biological relevance. However, allocation concealment, blinding, and sample-size justification were not clearly detailed, and the model remained treatment-interventional. |
| Fico and Santamaria-Martínez, 2020 | MMTV-PyMT;Tgfbi knockout mouse model assessing tumor hypoxia, CSC content, vascular normalization, immune compartment, tumor initiation, and metastasis | Unclear risk | Unclear risk | Unclear risk | Low risk | Unclear risk | Low to moderate concern | The genetic mouse model, tumor sphere assays, vascular and hypoxia assessments, metastasis analysis, and bone marrow transplantation strengthened biological validity. However, randomization, allocation concealment, and blinding safeguards were not fully reported. |
| Akbarpour et al., 2017 | TC1 murine epithelial lung tumor model exposed to intermittent hypoxia or sleep fragmentation, with CD8+ T-cell function and CSC marker assessment | Unclear risk | Unclear risk | Low to unclear risk | Low risk | Unclear risk | Low to moderate concern | The study used disease-relevant intermittent hypoxia and sleep fragmentation models, assessed tumor invasion and immune function, and reported blinded histological assessment. However, allocation concealment and full randomization procedures were not clearly detailed. |

**Supplementary Table S2. Observational quality assessment for human tissue, serum extracellular vesicle, or public dataset components**

| **Study** | **Human or dataset component assessed** | **Selection and representativeness** | **Comparability or control of confounding** | **Outcome or exposure measurement** | **Justification** |
| --- | --- | --- | --- | --- | --- |
| Wang et al., 2025 | Human TNBC tissue microarray assessing HIF-1α, MICA, CD56, Fzd7, and NK infiltration | Moderate concern | Moderate concern | Low concern | The human tissue component strengthened translational relevance, but representativeness and clinicopathological adjustment were limited. Outcome measurement was strengthened by multicolor immunohistochemistry and quantitative tissue analysis. |
| Dong et al., 2024 | Forty seven human glioma samples, public glioma datasets, single cell data, and spatial transcriptomic datasets | Low to moderate concern | Moderate concern | Low concern | The study combined institutional human samples with public datasets and spatial transcriptomic validation, improving external biological support. However, observational associations remain vulnerable to confounding and cannot establish causality. |
| Sami et al., 2020 | Human TNBC sample analysis linked to IRIS, immune suppression, TAMs, Tregs, CD8 cells, PD-L1, and CD47 | Moderate concern | Moderate concern | Low to moderate concern | Human sample validation supported the experimental findings, but selection methods and adjustment for clinical confounding were not fully detailed. The study was strengthened by integration with animal and mechanistic models. |
| Liu et al., 2022 | Ten human glioma tissues, TCGA glioma cohort, Ivy GAP, and immune infiltration analyses | Moderate to high concern | Moderate concern | Moderate concern | Public datasets strengthened biological interpretation, but the institutional tissue validation sample was small. The observational analyses support association between hypoxia, PGCCs, and M2 TAM signatures, but direct causal inference remains limited. |
| Hou et al., 2025 | NSCLC patient tissues and serum EVs assessing SNHG16 expression and association with clinicopathological features | Moderate concern | Moderate concern | Low to moderate concern | Human tissue and serum EV analysis strengthened translational relevance, but representativeness, adjustment for clinical confounding, and patient-level immune validation were limited. Outcome measurement was supported by molecular assays but remained mainly associative. |
| Jang et al., 2022 | TCGA and GEO pancreatic cancer survival analyses linked to CD44/SLC16A1 expression and stressed TME-derived cell phenotypes | Low to moderate concern | Moderate concern | Low to moderate concern | Public dataset analysis supported clinical relevance of CD44/SLC16A1-associated phenotypes, but the analysis remained observational and could not directly validate macrophage-to-cancer-cell transfer in patient tumors. |
| Fico and Santamaria-Martínez, 2020 | Human breast cancer tissue analysis and METABRIC/CIBERSORT assessment of TGFBI expression, prognosis, subtype association, myeloid/M2 enrichment, and CD8+ T-cell content | Low to moderate concern | Moderate concern | Low concern | Human tissue and METABRIC/CIBERSORT analyses strengthened translational interpretation, but associations between TGFBI, immune composition, prognosis, and aggressive subtypes remain vulnerable to confounding and cannot establish causality in patients. |

Newcastle–Ottawa-style domains were applied only to observational human tissue, serum extracellular vesicle, or public dataset components. Domain judgments were not combined into an overall rating. Studies using human-derived immune cells solely for ex vivo assays were treated as mechanistic rather than clinical observational components.

**Supplementary Table S3. Adapted methodological appraisal for in vitro and ex vivo mechanistic components**

| **Study** | **Mechanistic component assessed** | **Hypoxia exposure clarity** | **CSC characterization** | **Immune assay validity** | **Controls and comparators** | **Mechanistic validation** | **Main limitation** |
| --- | --- | --- | --- | --- | --- | --- | --- |
| Wang et al., 2025 | TNBC cell lines, TNBC CSC sorting, NK92MI and primary NK cytotoxicity, MICA-NKG2D axis, Fzd7 targeting | Low concern | Low concern | Low concern | Low concern | Low concern | Strong functional validation across hypoxia, CSC enrichment, NK cytotoxicity, and xenograft models, but translational inference remains preclinical. |
| Geng et al., 2024 | HT-29 derived colorectal cancer stem cells co cultured with NK cells under hypoxia, FADS1/DDA spermine axis | Moderate concern | Moderate concern | Low to moderate concern | Low to moderate concern | Low to moderate concern | The study used functional NK assays, cytokine analysis, apoptosis assays, and omics validation, but relied mainly on a single colorectal cancer cell line model without animal or human tumor validation. |
| Dong et al., 2024 | Glioma stem cells, U937 derived macrophages, TREM1, TLR2, AKT mTOR, TGF-β2, mesenchymal transition | Low concern | Low concern | Low to moderate concern | Low concern | Low concern | The mechanistic pathway was deeply validated across cell, tissue, dataset, and animal components, but macrophage modeling relied heavily on U937 derived macrophages, which may not fully represent primary TAM biology. |
| Sami et al., 2020 | IRIS overexpressing TNBC cells, GM-CSF, TAM recruitment, TGF-β1, CSC and EMT markers, CD47 and PD-L1 | Low concern | Low concern | Low concern | Low concern | Low concern | Strong multi model validation was provided, but the biology is centered on an IRIS overexpression system, which may limit generalizability across all TNBC contexts. |
| Hasmim et al., 2013 | B16 F10 melanoma hypoxia model, Nanog induction, TGF-β1 regulation, Treg and macrophage infiltration | Low concern | Low to moderate concern | Low concern | Low concern | Low concern | The study provides direct mechanistic evidence linking hypoxia induced Nanog to TGF-β1 mediated immunosuppression, but relies mainly on one melanoma model. |
| Bu et al., 2019 | Ovarian cancer spheroids, human derived CIK cells, HIF1A, ICAM 1, TGF-β1 SMAD and NF-κB signaling | Moderate concern | Low to moderate concern | Low concern | Low concern | Low concern | The study used ex vivo CIK cells from 20 donors and functional cytotoxicity assays, but depended mainly on A2780 spheroid models and chemical hypoxia induction. |
| Liu et al., 2022 | A172 glioma cells, CoCl₂ induced PGCCs, PGCC daughter cells, THP1 macrophage polarization, TCGA and tissue support | Moderate concern | Moderate concern | Moderate concern | Low to moderate concern | Moderate concern | The study links hypoxia induced PGCCs with stemness and M2 TAM polarization, but relies heavily on CoCl₂ induction, A172 cells, THP1 macrophages, and a small human tissue validation set. |
| Yin et al., 2022 | Endometrial cancer stem like cells, hypoxia and CoCl₂ induction, HIF-1α and HIF-2α mediated PD-L1 regulation | Low to moderate concern | Low concern | Moderate to high concern | Low concern | Low to moderate concern | The study strongly links hypoxia, PD-L1, and CSC self renewal, but direct immune effector functional assays were limited, so immune escape was mainly inferred through PD-L1 biology rather than tested through immune cell killing assays. |
| Hou et al., 2025 | Hypoxic NSCLC cell-derived SNHG16-containing EVs, A549 and H1299 cells, THP-1 macrophage polarization, SNHG16/miR-132-3p/KIF5A axis, CSC-related markers | Low concern | Moderate concern | Low to moderate concern | Low to moderate concern | Low to moderate concern | The study linked hypoxic EV signaling with CSC-related gene expression and M2 macrophage polarization, but CSC validation was mainly marker-based and the macrophage model relied on THP-1 cells rather than primary TAMs. |
| Liu et al., 2025 | Hypoxia-responsive HRA@D-TT delivery, 4T1/BCSC assays, 3D tumor penetration, CSC and MDSC differentiation, anti-PD-1 combination response | Low concern | Low to moderate concern | Low to moderate concern | Low concern | Low to moderate concern | The study provided strong interventional evidence targeting hypoxic CSC and MDSC compartments, but the mechanism was based on a therapeutic nanoplatform rather than direct endogenous CSC-immune crosstalk. |
| Zhang et al., 2025 | CuET/ICG nanoparticle-mediated mitochondrial interference, hypoxia relief, AMPK-mediated PD-L1 degradation on tumor cells and CSCs, T-cell killing, DC maturation, immune activation | Low concern | Low to moderate concern | Low concern | Low concern | Low concern | The study directly assessed CSC-associated PD-L1 and immune functional outcomes, but the evidence came from a treatment-reversal model with limited patient-level validation. |
| Jang et al., 2022 | Hypoxic and gemcitabine-stressed pancreatic cancer TME mimic, fibroblasts/CAFs, U937-derived macrophages, direct macrophage-to-cancer-cell transfer, CD44/SLC16A1 derivative cells | Low to moderate concern | Low to moderate concern | Moderate concern | Low to moderate concern | Moderate concern | The TME mimic and CSC functional assays were informative, but the immune component was macrophage-to-cancer-cell transfer rather than direct immune-effector function or immune suppression testing. |
| Fico and Santamaria-Martínez, 2020 | TGFBI-mediated vascular dysfunction, tumor hypoxia, CSC content, tumor sphere formation, metastatic CSCs, immune compartment analysis, myeloid/M2 and CD8+ associations | Low concern | Low concern | Moderate concern | Low concern | Low to moderate concern | The study strongly linked stromal/macrophage-associated TGFBI with hypoxia, CSC content, and metastasis, but immune evidence was mainly compositional rather than functional immune-effector testing. |
| Akbarpour et al., 2017 | Intermittent hypoxia and sleep fragmentation in TC1 tumor model, CD8+ T-cell cytotoxic function, GzmB, perforin, IFN-γ, CTL killing, Oct4+ and CD44+CD133+ CSC-like populations | Low concern | Moderate concern | Low concern | Low concern | Low to moderate concern | The study used a disease-relevant intermittent hypoxia model and functional CTL assays, but CSC assessment was mainly marker-based without sphere formation, self-renewal, or tumor-initiation assays. |
