# Supplementary material for: Hypoxic cancer stem cell–immune niches in the tumor microenvironment: a Systematic Review of mechanisms and therapeutic implications
Source: Front Immunol. 2026 Sep 22;17:1942424. doi: 10.3389/fimmu.2026.1942424 (PMC13638562; doi:10.3389/fimmu.2026.1942424)
Supplement: Supplementary file 4 [file Table4.docx]

**Supplementary Data Sheet 4. Reports excluded after full-text assessment and reasons for exclusion**

Each report was assigned one primary reason for exclusion according to the prespecified eligibility requirement that hypoxia or hypoxia-associated signaling, CSC or stemness phenotypes, and immune-related outcomes be evaluated within the same experimental or translational study. The category totals correspond to the PRISMA flow diagram.

| **No.** | **Report** | **DOI or identifier** | **Report-specific primary reason for exclusion** | **PRISMA exclusion category** |
| --- | --- | --- | --- | --- |
| 1 | Limantrafin-BODIPY Conjugates Reduce Stemness and Metastatic Potential via Superoxide Driven Phototherapy in Triple Negative Breast Cancer (2025) | 10.1002/smll.202505316 | Therapeutic phototherapy platform evaluated stemness and metastatic markers, including ICAM-1, but did not directly test a hypoxia-driven CSC–immune mechanism or functional immune outcome. | Therapeutic or platform-focused study without direct triad linkage |
| 2 | In situ injectable hydrogel encapsulating Mn/NO-based immune nano-activator for prevention of postoperative tumor recurrence (2024) | 10.1016/j.ajps.2024.100901 | Postoperative hydrogel/nano-activator study focused on therapeutic delivery and immune activation; it did not directly establish linked hypoxia, CSC, and immune mechanisms within the same system. | Therapeutic or platform-focused study without direct triad linkage |
| 3 | MiR-200c reprograms fibroblasts to recapitulate the phenotype of CAFs in breast cancer progression (2024) | 10.15698/cst2024.03.293 | Examined oxidative-stress-driven fibroblast reprogramming, treatment resistance, and immunosuppressive features, but did not directly test hypoxia-associated signaling, a defined CSC phenotype, and an immune mechanism as a linked triad. | No direct same-study CSC–immune mechanistic linkage |
| 4 | SND1, a novel co-activator of HIF1α, promotes tumor initiation in PyMT-induced breast tumor (2023) | 10.1111/febs.16940 | Evaluated HIF1α/SND1/EZH2 signaling and tumor initiation without an immune-cell, immune-function, checkpoint, or other eligible immune-modulation outcome. | No immune-related outcome relevant to immune modulation |
| 5 | A neutrophil-biomimic platform for eradicating metastatic breast cancer stem-like cells by redox microenvironment modulation and hypoxia-triggered differentiation therapy (2023) | 10.1016/j.apsb.2022.05.027 | Hypoxia-responsive drug-delivery platform targeted breast CSC differentiation, but the neutrophil membrane served as a biomimetic carrier rather than a functional immune component demonstrating CSC–immune crosstalk. | Therapeutic or platform-focused study without direct triad linkage |
| 6 | Targeting hypoxia-induced tumor stemness by activating pathogen-induced stem cell niche defense (2022) | 10.3389/fimmu.2022.933329 | The immune-related mechanism centered on pathogen-induced bystander apoptosis and stem-cell niche defense rather than tumor immune-cell modulation or antitumor immune dysfunction as defined by the review. | Non-cancer immune context |
| 7 | Quiescent cancer cells resist T cell attack by forming an immunosuppressive niche (2022) | 10.1016/j.cell.2022.03.033 | Characterized quiescent cancer-cell clusters and a local hypoxic immunosuppressive milieu, but quiescent cells were not operationally evaluated as CSC or stemness populations under the prespecified eligibility criteria. | No direct same-study CSC–immune mechanistic linkage |
| 8 | Hypoxia Contributes to Poor Prognosis in Primary IDH-wt GBM by Inducing Tumor Cells MES-Like Transformation Trend and Inhibiting Immune Cells Activity (2021) | 10.3389/fonc.2021.782043 | Relied on single-cell and bulk transcriptomic datasets to associate hypoxia, mesenchymal-like tumor states, and immune activity without experimental validation of the complete triad. | Correlative/bioinformatics-only evidence without experimental validation of the triad |
| 9 | Crucial role of glucosylceramide synthase in the regulation of stem cell-like cancer cells in B16F10 murine melanoma (2021) | 10.1002/mc.23347 | Investigated melanoma stem-like cells and immunomodulatory treatment, but did not directly evaluate hypoxia as the exposure or mechanism linking CSC and immune outcomes within the study. | No direct same-study CSC–immune mechanistic linkage |
| 10 | Nutritional supplements in combination with chemotherapy or targeted therapy reduces tumor progression in mice bearing triple-negative breast cancer (2021) | 10.1016/j.jnutbio.2020.108504 | Measured HIF-1α, CSC-associated markers, cytokines, and PD-1/PD-L1 as parallel treatment-response outcomes, but did not demonstrate a direct causal hypoxia–CSC–immune mechanistic linkage. | Therapeutic or platform-focused study without direct triad linkage |
| 11 | Development and validation of a novel stem cell subtype for bladder cancer based on stem genomic profiling (2020) | 10.1186/s13287-020-01973-4 | Computational genomic subtyping associated stemness, hypoxia, immune infiltration, and predicted immunotherapy response without experimental validation of the linked triad. | Correlative/bioinformatics-only evidence without experimental validation of the triad |
| 12 | EMP2 is a novel regulator of stemness in breast cancer cells (2020) | 10.1158/1535-7163.MCT-19-0850 | Evaluated EMP2 regulation of breast CSC phenotypes but did not concurrently assess hypoxia and an eligible immune-modulation mechanism. | No direct same-study CSC–immune mechanistic linkage |
| 13 | Altered molecular pathways decides the treatment outcome of Hsp90 inhibitors against breast cancer cells (2020) | 10.1016/j.tiv.2020.104828 | Treatment-response study of Hsp90 inhibition and tumor-cell pathways did not directly evaluate the complete hypoxia–CSC–immune triad or functional immune modulation. | Therapeutic or platform-focused study without direct triad linkage |
| 14 | C/EBPδ links IL-6 and HIF-1 signaling to promote breast cancer stem cell-associated phenotypes (2019) | 10.1038/s41388-018-0516-5 | Linked HIF-1/IL-6 signaling to tumor-cell stemness, but did not directly evaluate an immune-cell or immune-function outcome linking CSC biology to immune modulation. | No direct same-study CSC–immune mechanistic linkage |
| 15 | Ocoxin Modulates Cancer Stem Cells and M2 Macrophage Polarization in Glioblastoma (2019) | 10.1155/2019/9719730 | Evaluated CSC properties and macrophage polarization after Ocoxin treatment, but hypoxia or hypoxia-associated signaling was not a central exposure or directly assessed mechanism. | Hypoxia not central or not directly assessed as a primary exposure/mechanism |
| 16 | Obesity promotes the expansion of metastasis-initiating cells in breast cancer (2018) | 10.1186/s13058-018-1029-4 | Associated obesity-related hypoxia and a neutrophil-rich microenvironment with metastasis-initiating cells, but did not directly establish a same-study mechanistic CSC–immune interaction. | No direct same-study CSC–immune mechanistic linkage |
| 17 | A 3D model of tumour angiogenic microenvironment to monitor hypoxia effects on cell interactions and cancer stem cell selection (2017) | 10.1016/j.canlet.2017.03.006 | Modeled hypoxia, angiogenic/stromal interactions, and CSC selection without an eligible immune-cell or immune-function outcome. | No immune-related outcome relevant to immune modulation |
| 18 | Exposure of tumor-associated macrophages to apoptotic MCF-7 cells promotes breast cancer growth and metastasis (2015) | 10.3390/ijms160611966 | Demonstrated macrophage–CSC crosstalk after exposure to apoptotic tumor cells, but hypoxia was not the primary exposure or directly tested mechanism. | Hypoxia not central or not directly assessed as a primary exposure/mechanism |
| 19 | Depletion of carcinoma-associated fibroblasts and fibrosis induces immunosuppression and accelerates pancreas cancer with reduced survival (2014) | 10.1016/j.ccr.2014.04.005 | Fibroblast depletion produced hypoxia, CSC features, and immunosuppression, but hypoxia was a downstream accompanying phenotype rather than the primary exposure or directly tested coordinating mechanism. | Hypoxia not central or not directly assessed as a primary exposure/mechanism |
| 20 | Developing a Protocol for In Vitro Characterization of Circulating Cancer Stem Cell-like Cells (2024) | GSI-2024-00018 (doctoral thesis) | Method-development thesis/protocol for characterizing circulating CSC-like cells; it did not provide eligible primary evidence linking hypoxia, CSC phenotype, and tumor immune modulation. | No direct same-study CSC–immune mechanistic linkage |
| 21 | miR-205 hinders the malignant interplay between prostate cancer cells and associated fibroblasts (2014) | 10.1089/ars.2013.5292 | Examined HIF-1-associated oxidative/pro-inflammatory tumor–fibroblast crosstalk and EMT, but did not concurrently validate a CSC phenotype and an eligible immune-related outcome. | No direct same-study CSC–immune mechanistic linkage |
| 22 | HIF1A expression correlates with increased tumor immune and stromal signatures and aggressive phenotypes in human cancers (2020) | 10.1007/s13402-020-00534-4 | Pan-cancer TCGA analysis correlated HIF1A expression with stemness and immune/stromal signatures without experimental validation of the complete triad. | Correlative/bioinformatics-only evidence without experimental validation of the triad |
| 23 | Identification of a NACC1-Regulated Gene Signature Implicated in the Features of Triple-Negative Breast Cancer (2023) | 10.3390/biomedicines11041223 | Gene-signature and dataset analyses associated TNBC stemness with hypoxia and immune-suppressive features, but did not experimentally validate linked hypoxia–CSC–immune mechanisms. | Correlative/bioinformatics-only evidence without experimental validation of the triad |
| 24 | The landscape of malignant transition: Unraveling cancer cell-of-origin and heterogeneous tissue microenvironment (2025) | 10.1016/j.canlet.2025.217591 | Broad pan-tissue atlas of malignant transition and microenvironment heterogeneity; it did not directly test the hypoxia–CSC–immune triad within a defined experimental or translational system. | No direct same-study CSC–immune mechanistic linkage |
| 25 | Analysis of Single-Cell RNA-Seq Data to Investigate Tumor Cell Heterogeneity in Uroepithelial Bladder Cancer and Predict Immunotherapy Response (2026) | 10.2174/0115680096377593250626133719 | Single-cell and bulk-data analysis linked stemness with predicted immunotherapy response but did not experimentally validate hypoxia-driven CSC–immune mechanisms. | Correlative/bioinformatics-only evidence without experimental validation of the triad |

**Category totals:** No direct same-study CSC–immune mechanistic linkage (n = 9); Therapeutic or platform-focused study without direct triad linkage (n = 5); Hypoxia not central or not directly assessed as a primary exposure/mechanism (n = 3); No immune-related outcome relevant to immune modulation (n = 2); Correlative/bioinformatics-only evidence without experimental validation of the triad (n = 5); Non-cancer immune context (n = 1).
